# Randomised feasibility study evaluating eye movement desensitisation and reprocessing therapy for functional neurological disorder (MODIFI)

**DOI:** 10.1007/s00415-025-13219-5

**Published:** 2025-07-08

**Authors:** Sarah R. Cope, Jared G. Smith, Sharif El-Leithy, Serena Vanzan, Patricia Hogwood, Dawn Golder, Kati Jane Turner, Maeve Crowley, Jo Billings, Susannah Pick, Caitlin Pentland, Mark J. Edwards

**Affiliations:** 1 Clinical Research Unit, South West London and St George’s Mental Health Trust, Springfield University Hospital, 61 Glenburnie Road, London, SW17 7DJ UK; 2 Department for Clinical Neuropsychology and Clinical Health Psychology, St George’s University Hospitals Foundation NHS Trust, Blackshaw Road, London, SW17 0QT UK; 3 Inpatient Neuropsychiatry Services, South London and Maudsley NHS Foundation Trust, Maudsley Hospital, Denmark Hill, London, SE5 8AZ UK; 4 Population Health Research Institute, St George’s, University of London, Cranmer Terrace, Tooting, London, SW17 0RE UK; 5 Traumatic Stress Service, South West London and St. George’s Mental Health NHS Trust, Springfield University Hospital, 61 Glenburnie Rd, London, SW17 7DJ UK; 6 Sussex Partnership NHS Trust, Portland House, Richmond Road, Worthing, West Sussex BN11 1HS UK; 7 Department of Psychiatry, University College London, Wing A, 4 Floor, Maple House, 149 Tottenham Court Road, London, W1T 7NF UK; 8 Department of Psychological Medicine, Institute of Psychiatry, Psychology and Neuroscience, King’s College London, London, SE5 8AF UK; 9 Department of Basic and Clinical Neuroscience, Institute of Psychiatry, Psychology and Neuroscience, King’s College London, London, SE5 8AF UK

**Keywords:** Functional neurological disorder, Functional motor disorder, Functional seizures, Psychological therapy, Eye movement desensitisation and reprocessing therapy, Randomised controlled trial

## Abstract

**Background:**

Functional neurological disorder (FND) is a common neurological presentation with symptoms such as seizures, walking difficulties, limb weakness and cognitive difficulties. Treatments for FND include physiotherapy and psychological therapy. Eye movement desensitisation and reprocessing therapy (EMDR) is a therapy designed to reduce disturbance associated with distressing or traumatic memories. Case report evidence suggests possible benefit for people with FND. This randomised feasibility study aimed to assess whether a large-scale trial evaluating EMDR for FND would be feasible and acceptable.

**Methods:**

Fifty participants with FND were randomised to either FND-focused EMDR plus standard neuropsychiatric care (NPC) or NPC alone. Feasibility criteria were recruitment rate, intervention adherence, and outcome measure completion. Assessment of safety was also examined, as well as therapy satisfaction. Participants completed questionnaires at baseline, 3 months, 6 months and 9 months. FND symptoms were assessed using Ecological Momentary Assessment at each time point.

**Results:**

Recruitment rate was 58%, intervention adherence was 88%, and outcome measure completion was 68% for Ecological Momentary Assessment and 76% for questionnaires at 9-month follow-up. Participants experienced functional motor symptoms (80%), functional seizures (64%), and cognitive symptoms (32%). Participants receiving EMDR + NPC reported greater satisfaction and greater FND improvement compared to NPC. Questionnaire data suggested greater reductions in PTSD, depression, anxiety, dissociation, disability and healthcare-use for EMDR + NPC.

**Discussion:**

The study demonstrated that an FND-specific protocol for EMDR was feasible and acceptable. Potential positive effects on FND symptoms, mental health, disability, and healthcare utilisation were found. A full-scale trial is warranted to establish efficacy.

**Trial Registration:**

NCT05455450 (www.clinicaltrials.gov).

**Supplementary Information:**

The online version contains supplementary material available at 10.1007/s00415-025-13219-5.

## Introduction

Functional neurological disorder (FND) is a condition at the interface between neurology, psychiatry, and psychology [1]. It is characterised by genuine motor, sensory, and perceptual changes that can cause significant distress and impairments in functioning. Presenting symptoms can include motor symptoms such as gait disturbance, jerking of limbs, and tremor, seizure-like episodes (functional seizures), cognitive symptoms such as brain fog and memory impairments, sensory symptoms such as numbness or tingling, dizziness (persistent postural perceptual dizziness) and speech or swallowing difficulties [2]. FND is commonly associated with comorbid conditions, e.g. chronic pain, persistent fatigue, irritable bowel syndrome, anxiety disorders, post-traumatic stress disorder (PTSD), depression, emotional instability, autistic spectrum disorder, and dissociative symptoms [2–5]. FND is also associated with altered emotion and interoceptive processing, insecure attachment styles, and alexithymia [6–9]. People with FND are more likely to have experienced adverse events in childhood and adulthood compared to healthy controls [10]. Adverse life events (including injury or illness) are associated with the triggering of FND symptoms [11, 12]. FND is more prevalent than many other neurological disorders [13]. Untreated FND is associated with high healthcare costs through repeated referrals and investigations, inpatient admissions and emergency department attendances [14, 15], as well as other life costs relating to disability and unemployment [16, 17].

FND presentations are heterogenous, with a range of underlying biopsychosocial processes, and patients frequently present with more than one functional neurological symptom; therefore, different therapies may suit different patients. Treatment for FND includes effective communication of the diagnosis [18–21]. Basic education regarding understanding FND and coping with symptoms (e.g. distraction strategies) can be delivered individually or in a group format [20, 22]. There is good evidence for physiotherapy at treating functional motor symptoms, but physiotherapy on its own is not suitable for the majority of patients [23, 24]. Multi-disciplinary treatment (including neurology, psychiatry, physiotherapy, clinical psychology, occupational therapy, speech and language therapy) is recommended for more complex FND presentations, and there is evidence of benefit from inpatient and outpatient multi-disciplinary treatment programmes [19, 25–28]. In terms of psychological therapy, there is some evidence of benefit, particularly for functional seizures, with the highest quality of evidence for cognitive–behavioural therapy; although psychodynamic and other therapies have also reported positive outcomes in uncontrolled studies [29–33].

EMDR therapy is an established treatment for PTSD, but it is a therapy that can help with any upsetting memories or images. It is an integrative therapy that includes aspects of psychodynamic, cognitive–behavioural, experiential, and somatic therapies. It follows a standard eight-phase protocol. The therapy is based on the adaptive Information processing model, which suggests that traumatic or adverse experiences can disrupt normal information processing and inhibit the brain’s innate ability to process traumatic experiences. This “imbalance” in the system leads to unhelpful behaviours and beliefs, and can contribute to unwanted symptoms. EMDR therapy is proposed to work by facilitating the brain’s natural ability to process distressing information and access adaptive material. This adaptive information processing can then lead to symptom reduction, e.g. reduced intrusive memories or flashbacks to traumatic material in the context of PTSD, and/or reduced associated somatic sensations once relevant memories have been processed [34]. EMDR therapy traditionally uses eye movements as a form of “bilateral stimulation”, but other types of alternate bilateral tasks can also be used effectively, e.g. listening to bilateral audio tones, or tapping alternately. Bringing a memory to mind, whilst engaging in a competing task like side-to-side eye movements, can reduce vividness and emotionality associated with a memory and lead to reduced symptoms [35].

We propose that EMDR may be a beneficial psychological therapy for FND for these reasons:

1. FND is often associated with adverse events prior to symptoms, as well as higher rates of traumatic experiences across the lifetime. EMDR therapy can focus on memories of major trauma (significant violence, sexual assault, life-threatening accident) as well as distressing experiences (bullying, relationship discord, work stress), including distressing memories of having FND [36]. Therefore, EMDR therapy can be flexibly applied, not just for those with experiences of major trauma.
2. EMDR therapy focuses on reprocessing key memories, decreasing somatic and emotional arousal, resulting in reduced re-experiencing of associated physical sensations, emotions and cognitions [34]. Reducing distress associated with key memories or predictive images associated with FND could theoretically weaken the internal representations of symptoms (influencing abnormal “priors”), as well as reducing threat associated with symptoms, therefore also potentially reducing the likelihood that these representations are activated (resulting in less FND symptoms);
3. FND is associated with alterations in interoception, and alexithymia. The inclusion of the body as a focal point within EMDR therapy can increase awareness and understanding of connections between emotions and the body [37].
4. Adverse childhood experiences (ACEs) are associated with greater risk of illness in later life [38]. ACEs can cause disruption in the development of the nervous, endocrine, immune and metabolic systems [39]. ACEs can also impact negatively on how a person learns to cope with stressors, e.g. using avoidant emotion-focused strategies, rather than problem-focused strategies [40, 41]. Given the higher likelihood of ACEs and FND [42], and the association between impaired emotional processing and FND, therapy like EMDR that focuses on enhancing emotional processing, and memories of traumatic experiences, could be of benefit.
5. PTSD rates are higher in FND populations (particularly functional seizures), and EMDR is a therapy with good efficacy for treating PTSD [42, 43].
6. Trauma-focused treatment for PTSD can reduce dissociative symptoms, including somatoform dissociation [44–47].
7. There is increasing evidence EMDR is efficacious at treating non-PTSD conditions, e.g. chronic pain, tinnitus, depression.[48–51]. EMDR can be used to reduce distress associated with a diagnosis, its consequences, and/or experiences in medical settings [52]. For example, in the case of chronic pain, EMDR can focus on any traumatic event(s) associated with the pain, traumatic consequence(s) of the pain, and/or the pain symptoms themselves in session [53].
8. There is evidence of effectiveness in treating functional or “medically unexplained” symptoms generally [53], including case report evidence in FND [54, 55].

We, therefore, planned a study to assess the feasibility and acceptability of conducting a full-scale trial of EMDR for FND. An FND-specific EMDR treatment protocol was used. Feasibility was assessed via recruitment rate, intervention adherence, and retention. Attendance rates, satisfaction ratings, therapy fidelity ratings, and qualitative interviews were used to assess acceptability. Adverse events across both arms were measured. Completion of outcome measures, the intervention itself, and the results were reviewed and will be used to inform the design of a future full-scale trial.

## Methods

The study followed the method described in the published protocol and is in accordance with the CONSORT reporting guidelines [56, 57].

### Research ethical approval

The research was reviewed by the NHS West Midlands—Edgbaston Research Ethics Committee with a favourable opinion (Reference: 22/WM/0178), and Health Research Authority approval was received (both dated 27th September 2022).

### Study design

This feasibility study was a single-blind randomised controlled trial (RCT) with two arms: EMDR (plus standard neuropsychiatric care (NPC)) and standard NPC only. The two groups were compared at baseline, 3 months, 6 months, and 9 months. We recruited fifty adult patients with a diagnosis of FND (confirmed by a neurologist according to standardised diagnostic criteria), from a single site (neuropsychiatry service in the UK). After participants had completed their intervention period, a selection of them were invited to take part in semi-structured interviews regarding their experiences of the trial. Participants were invited to take part in interviews if they had completed their time in the trial and if they had been randomised to EMDR + NPC. Additionally, participants randomised to NPC were invited to be interviewed, once they had completed their time in the trial, and they were selected using purposive sampling to ensure a balance of symptom presentations. The trial therapists were also interviewed.

### Allocation and blinding

Participants were randomised into EMDR + NPC or NPC alone in a 1:1 ratio. A stratified block randomisation (using randomly permuted blocks of sizes 2 and 4) was used so that there were similar numbers of participants with and without PTSD symptoms in each arm (PTSD diagnosis determined by diagnostic algorithm of the International Trauma Questionnaire). Randomisations were carried out by the Trial Manager (SV) using the randomisation function on REDCap.

The research assistant and statistician remained blind to treatment allocation.

### Public and patient involvement (PPI)

PPI has been present in the research design process, throughout the delivery of the trial (PPI representatives within the Trial Management Group and Trial Steering Committee), and in contributions to trial materials, interview schedules, and publications.

### Recruitment

Potential participants who expressed an interest in taking part in FND research were referred to the research team by the Neuropsychiatry service. The research assistant gave potential participants a summary of the study, the Participant Information Sheet and Informed Consent Form to review. Those willing to participate gave informed consent and were scheduled for a screening interview.

The screening interview was used to establish eligibility in terms of inclusion criterion (5) Reporting at least one traumatic event on the International Trauma Exposure Measure (ITEM), and exclusion criterion (7) Diagnosis of dissociative identity disorder or score in clinical range on “identity disturbance” subscale of Multiscale Dissociation Inventory (MDI). Potential participants needed to score in the non-clinical range of the subscale “Identity Disturbance” on the MDI to take part (score < 15). Eligible participants completed additional baseline measures, and provided demographic information, medical history, and previous psychological therapies attended.

### Incentives

Participants were offered reimbursement for research-related travel costs (up to £20 per appointment). A non-contingent £25 incentive was offered to participants 9 months after informed consent, unrelated to trial completion. All participants who completed an interview were offered a £20 incentive.

### Eligibility criteria

Inclusion criteria were (1) predominant diagnosis of functional seizures and/or functional motor symptoms, with diagnosis confirmed by neurologist; (2) aged 18 years or over; (3) capacity to consent; (4) willingness to attend regular psychological therapy sessions; (5) reporting at least one traumatic event on the International Trauma Exposure Measure (ITEM).

Exclusion criteria were (1) non-english speaking; (2) current ongoing adversity that is likely to interfere with psychological therapy, e.g. domestic violence, homelessness, unresolved compensation claim/litigation; (3) predominant diagnosis of borderline personality disorder*; (4) predominant diagnosis of chronic pain condition*, e.g. fibromyalgia; (5) predominant diagnosis of chronic fatigue syndrome*; (6) diagnosis of a psychotic disorder; (7) diagnosis of dissociative identity disorder or score in clinical range on “identity disturbance” subscale of MDI; (8) uncontrolled epileptic seizures; (9) diagnosis of an eating disorder; (10) current severe self harm or strong suicidal ideation that requires secondary care mental health services input; (11) current alcohol or drug harmful use or dependence; (12) current diazepam use exceeding the equivalent of 10 mg per day; (13) currently attending individual psychological therapy focused on FND or other specialist FND-specific treatment such as inpatient/outpatient multi-disciplinary treatment or intensive FND-specific physiotherapy.

*Comorbid diagnosis was acceptable, as long as FND was the predominant difficulty.

### Study interventions

#### *EMDR plus standard neuropsychiatric care (EMDR* + *NPC)*

Participants randomised to EMDR + NPC were offered up to sixteen EMDR sessions, (completed within six months), a one-month optional follow-up session (not counted as an EMDR session), and standard outpatient neuropsychiatric appointments (NPC). Participants could choose to attend 60–90 min sessions either in-person or virtually via MS Teams. A minimum of 8 sessions counted as completed treatment.

The standard EMDR protocol was delivered, but was adapted for FND presentations, following a treatment protocol developed by SC. Treatment has three stages: 1. Assessment, psychoeducation, target selection, and preparation for processing; 2. Processing of targets; 3. Ending of therapy. The initial sessions included education on FND, anxiety and dissociation; as well as formulating collaboratively with the participant regarding their FND symptoms’ development and maintenance. With the participant, target memories/images were chosen, such as (1) distressing memories associated with the time when symptoms began or FND symptoms generally; (2) distressing memories from past events that may be relevant to their FND symptoms; (3) FND symptoms themselves, when present in session, or an image of them; (4) distressing images about the future, e.g. image of having FND symptoms in front of others.

#### Standard neuropsychiatric care (NPC)

NPC was standard care and included 1–3 routine out-patient appointments with a neuropsychiatrist during the trial period. Participants were still invited to psycho-educational group interventions focused on FND routinely administered by the service. Their neuropsychiatrist referred for psychological therapy outside of the service for any comorbid conditions, but not for FND-specific psychological therapy or other therapies.

### Training, supervision and fidelity checks

There were two trial therapists who had both completed EMDR-Europe Accredited Basic EMDR training (Parts 1–3): one trial therapist was a clinical psychologist who had completed their EMDR training in July 2021 (around 18 months post-training experience before trial). The other trial therapist was a cognitive–behavioural therapist who had completed EMDR training in April 2019 (around 44 months post-training experience before trial). Neither were accredited EMDR therapists yet, so can be considered relatively novice EMDR therapists. They both attended training on the FND-specific EMDR protocol. They received clinical supervision from SC (clinical psychologist and accredited EMDR Therapist), as well as external EMDR supervision from a clinical psychologist and EMDR Consultant (MC), with both supervisions every 2–4 weeks. Therapists completed a session record form after every session.

All sessions of EMDR were video-recorded (with the participants’ consent) and excerpts were shown in EMDR supervision. Fifteen randomly selected recordings of processing sessions were rated for fidelity by independent EMDR Consultants, using the EMDR Fidelity Rating Scale Version 2–Adverse Life Experiences Processing subscale [58].

### Primary objectives and outcome measures

A mixed methods approach was used to establish feasibility and acceptability. The feasibility criteria were recruitment rate (% potentially eligible participants attending screening interview), intervention adherence (% participants randomised to EMDR + NPC who complete therapy), and outcome measure completion (% participants who complete outcome measures at all time points), with a target of above 70%, and a cut-off of above 50% demonstrating feasibility. If any criteria fell into the 50–70% bracket, ways to improve outcome(s) were considered [57].

Assessment of safety (adverse/serious adverse events) was examined between the two arms. Therapy satisfaction, choice of therapy session format (in-person/virtual), and therapy fidelity were No formal hypothesis testing of the feasibility datae evaluated and required sample size calculated to consider a primary outcome for a substantive RCT.

The nested qualitative study using interviews from participants and trial therapists will inform the substantive study. Participants’ qualitative experiences of the trial will be reported in another paper.

#### Outcome measures

Ecological Momentary Assessment using the m-Path App was used to measure FND symptoms [59]. This measurement approach was chosen as there is currently no single outcome measure that measures the variety and variability of FND symptoms [60]. Participants chose a maximum of two symptoms at the beginning of the trial period to rate, e.g. seizures, tremor, limb weakness, tingling/numbness, gait disturbance. They rated their chosen symptom(s) daily for two weeks at Baseline, and 3 months, 6 months, and 9 months follow-ups. They answered five questions each day in reference to their chosen symptom(s) (frequency, severity, interference, associated distress, associated preoccupation).

Other outcome measures were World Health Organisation Disability Assessment Schedule (WHODAS 2.0), EQ-5D-5L, PHQ-9, GAD-7, International Trauma Exposure Measure (ITEM), International Trauma Questionnaire (ITQ), Multiscale Dissociation Inventory (MDI), Adult Service Use Schedule (AD-SUS), Clinical Global Impression – Improvement Scale (CGI-I) rated by participant (single item, 7-point scale), and CGI-I rated by person nominated by participant (e.g. family member, partner, close friend, carer) (single item, 7-point scale) [61–69]. All the previously listed measures were completed at Baseline, 3 months, 6 months, and 9 months, except for the ITEM (Baseline only), the AD-SUS (Baseline and 9 months only), and the CGI-I (9 months only). Participants were also asked questions regarding ‘Agreement with diagnosis of FND’ (single item, 11-point scale), ‘Preference regarding treatment’ (EMDR + NPC, NPC or no preference), and ‘Belief in having been given the right treatment’ (single item, 11-point scale) at Baseline and 9 months. The satisfaction rating of treatment (single-item, 11-point scale) was asked at 9 months follow-up. Detailed descriptions of outcome measures and the schedule of assessments for participants can be found in the protocol paper [57].

### Statistical analysis

This was a feasibility trial, and as such, a power calculation was not performed. Rather, a target recruitment of 50 participants was considered sufficient to examine the feasibility of a subsequent definitive RCT as a function of recruitment, adherence and retention rates [70]. A baseline table compared the demographic and key clinical characteristics between the two trial arms in a descriptive manner, summarising data by frequency (%) for categorical variables and mean (SD) for continuous variables that followed a Gaussian distribution and median (inter-quartile range) otherwise. Feasibility outcomes were summarised using descriptive statistics and compared to full-trial progression criteria, and acceptability of the intervention was evaluated primarily via examination of satisfaction of care, treatment preference and clinical global impression data at 9-month follow-up. Descriptive assessments of EMDR session characteristics (e.g., number/length of sessions, fidelity to EMDR protocol) and health care resource use stratified by treatment arm were also considered.

Analyses conducted to explore potential effects of EMDR therapy at 3-, 6- and 9-month follow-ups were, in the first instance, based on intention-to-treat (ITT) principles: all randomly assigned patients enrolled in the MODIFI trial who completed baseline measures were included. Each dependent variable was analysed by fitting Generalized Linear Mixed Models (GLMM), which allow for different numbers of repeated measurements between participants and response variables from different distributions, accounts for within-person clustering of data, and is appropriate for evaluation of intensive longitudinal data such as Ecological Momentary Assessment (EMA) [71, 72]. For individuals reporting functional seizures as one of their two symptoms, GLMM with linear (with identity link) and binomial (with logit link) distributions were respectively used to evaluate the impact of the intervention on EMA seizure frequency (transformed using Box–Cox methods to reduce positive skew) and on (the proportion of) days with at least one seizure; these models included condition (EMDR + NPC and NPC), time (baseline, 3-, 6-, and 9-month follow-ups) and condition*time (interaction) as fixed effects, and random intercepts at symptom (1 and 2), time, day (1–14), and person (subject-specific) levels. To assess treatment-specific changes in FND symptom severity, interference, distress and preoccupation (across all participants), four separate GLMM with linear distribution were administered; these included condition, time, symptom type (functional seizure, functional motor, and functional cognitive), symptom (1 and 2) and condition*time and condition*time*symptom type (interactions) as fixed effects and (as above) random intercepts at symptom (1 and 2), time, day (1–14), and person (subject-specific) levels. Inclusion of the condition*time*symptom interaction term in each model allowed the evaluation of any potential differential effects of the trial intervention according to (broad) FND symptom class (in addition to gauging an overall effect). GLMM with linear distribution was also employed to examine signal of efficacy for constructs assessed at each time point by standardised questionnaires (e.g., PTSD symptoms, disability levels, HRQoL and anxiety); these random intercept models included condition, time and condition*time interaction as fixed effects. Exploratory analyses (separately) considered potentially differential patterns of change in participants according to the presence of PTSD at pre-treatment and (for standardised questionnaire outcomes) whether the participants experienced functional seizures (by adding main effect and corresponding interactive fixed effects to GLMM).

All GLMM used restricted maximum likelihood (REML) estimation, which produces unbiased estimates in case of small sample sizes and employed first-order autoregressive (or first-order autoregressive moving average) structures for the residuals to account for time dependencies between adjacent assessments [73]. Where distribution of model residuals significantly differed from normality, dependent measures were transformed to better approximate a Gaussian distribution (e.g., EQ-5D-5L total value). Missing data were assumed to be missing at random (the least restrictive assumption); findings from Little’s Test of Missing Completely at Random supported this (for all tests, *p* > 127) [74].

Subsequent ‘per-protocol’ analyses, considering only those participants in the EMDR + NPC group completing treatment (i.e., attending ≥ 8 sessions) and with data at the time-point for the relevant measure (for EMA, this necessitated responses for no less than 5 of the 14 days in the time period) were administered; analysis of EMA data were performed in the manner above (i.e., fitting GLMM), while for standardised questionnaire outcome measures, between-group differences for primary and secondary outcome measures at each follow-up time-point, adjusted for pre-treatment score on the measure of interest, were calculated using analysis of covariance (ANCOVA), which relies on complete-case analysis).

Estimates of potential intervention effects (Hedge’s g) were calculated in GLMM by dividing the difference in change between the two trial arms (from estimated marginal means) at all post-randomisation time points by the corresponding pre-treatment pooled SD [75], and in per-protocol analyses by dividing the difference between (adjusted) mean scores at the time point by its pooled SD. An effect size of 0.2 was considered a small effect, 0.5 as a moderate effect and 0.8 as a large effect [76]. Interval estimates of these effects were produced in the form of 95% confidence intervals (CI), to explore imprecision and ensure the effect size subsequently chosen for powering a definitive trial is plausible [77]. No formal hypothesis testing of the feasibility data was undertaken.

Statistical analyses were undertaken using Statistical Package for the Social Sciences (SPSS) Version 29 (IBM Corporation, 2022), supplemented where required by Stata SE Version 16.0.

## Results

### Trial recruitment and acceptability

#### Recruitment

**Fig. 1:**
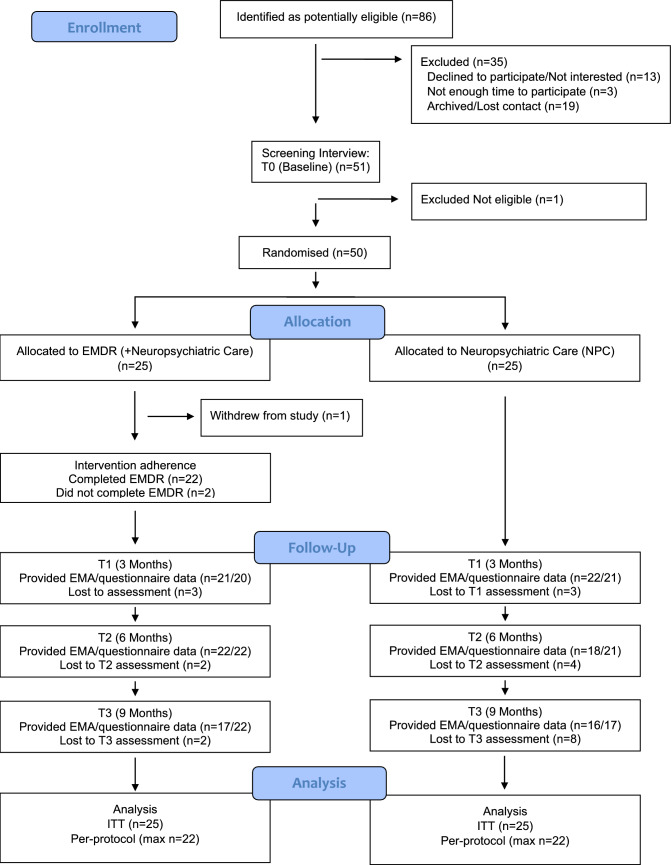
Consolidated Standards of Reporting Trials (CONSORT) Diagram for Participant Note: ITT = Intention-to-Treat. Per-protocol (max n) = maximum number of participants at any of the follow-ups for the EMDR (plus NPC) condition was n = 22

Participants were recruited from 12 December 2022 to 11 November 2023. The study was closed after n = 50 participants had been randomised into EMDR + NPC (n = 25) or NPC (n = 25). Participant flow is shown in Fig. 1. Clinicians in recruiting clinics referred 86 patients with FND to the trial and provided information about the study (71 (82.6%) from an initial assessment clinic, 15 (17.4%) from a follow-up clinic). Fifty-one consented to participate and were formally assessed for eligibility at a screening interview; 50 were found to be eligible and were randomised, equalling to a participation rate of 58.1% (CI = 47.0%,68.7%). Sociodemographic and clinical characteristics of ineligible and non-consenting patients were not available; as such, comparisons with consenting participants were not possible.

### Baseline characteristics

Table 1 shows baseline sociodemographic and clinical characteristics according to trial condition. The mean age was approximately 30 years. Most participants were women, white (British) and single (or separated/widowed), with approximately equal distribution between conditions. A little over half were working or in education while a similar proportion were in receipt of state benefits, although the latter was more common for participants in the NPC condition.

**Table 1:** Baseline demographic and clinical characteristics of the intention-to-treat participants in EMDR (+ NPC; n = 25) and NPC (n = 25) conditions

	EMDR + NPC	NPC	Total
Age (years; med, IQR)	32.4 (22.3,39.6)	34.1 (26.6,46.1)	32.7 (24.1,43.0)
Gender			
*Female*	21 (84.0)	18 (72.0)	39 (78.0)
*Male*	2 (8.0)	4 (16.0)	6 (12.0)
*Other/Prefer not to say*	2 (8.0)	3 (12.0)	5 (10.0)
Ethnicity			
*White British*	19 (76.0)	18 (72.0)	37 (74.0)
*White Irish/Other*	2 (8.0)	3 (12.0)	5 (10.0)
*Black/Black British*	2 (8.0)	2 (8.0)	4 (8.0)
*Dual Heritage*	2 (8.0)	2 (8.0)	4 (8.0)
Relationship status			
*Married or living with partne*r	8 (32.0)	9 (36.0)	17 (34.0)
*Single, separated, or widowed*	16 (64.0)	16 (64.0)	32 (64.0)
*Not disclosed*	1 (4.0)	0 (0.0)	1 (2.0)
Currently employed or in education	15 (60.0)	11 (44.0)	26 (52.0)
Receipt of state benefits	9 (36.0)	18 (72.0)	27 (54.0)
Has dependents	4 (16.0)	12 (48.0)	16 (32.0)
Presence of carer	4 (16.0)	7 (28.0)	11 (22.0)
Time since FND symptoms onset			
*Previous year*	5 (20.0)	8 (32.0)	13 (26.0)
*2–3 years*	10 (40.0)	10 (40.0)	20 (40.0)
*≥ 4 years*	10 (40.0)	7 (28.0)	17 (34.0)
Time since FND diagnosis			
*Same or previous year*	19 (76.0)	17 (68.0)	36 (72.0)
*≥ 2 years*	6 (24.0)	8 (32.0)	14 (28.0)
Beliefs			
*Agreement with FND diagnosis (0–10;med,IQR)*	9.0 (7.0,10.0)	10.0 (8.0,10.0)	9.0 (7.8,10.0)
Preference regarding treatment of your FND			
*EMDR + NPC*	16 (64.0)	18 (72.0)	34 (68.0)
*NPC*	1 (4.0)	1 (4.0)	2 (4.0)
*No preference*	8 (32.0)	6 (24.0)	14 (28.0)
Functional symptoms			
*Motor symptoms*	20 (80)	20 (80)	40 (80)
*Cognitive symptoms*	9 (36)	7 (28)	16 (32)
*Seizures*	16 (64)	16 (64)	32 (64)
*Weekly seizure frequency (mean, SD)*	8.1 (12.8)	11.6 (13.4)	9.8 (13.0)
*% days over 2 weeks with ≥ 1seizure (mean, SD)*	37.5 (34.5)	50.1 (35.4)	43.8 (34.9)
Trauma			
*PTSD criteria met (ITQ)*	9 (36.0)	11 (44.0)	20 (40.0)
*Complex PTSD criteria met (ITQ)*	8 (32.0)	7 (28.0)	15 (30.0)
Traumatic events (ITEM; 0–66; mean, SD)	8.64 (6.64)	8.16 (5.24)	8.40 (5.93)
Traumatic life events (Crit.A; 0–48; mean., SD)	4.32 (3.40)	3.88 (2.60)	4.10 (3.01)
Psyc. threatening events (Crit.B; 0–18; mean, SD)	4.32 (3.61)	4.28 (3.58)	4.30 (3.56)
Mood			
*PHQ-9 Moderate/Severe*	21 (84.0)	22 (88.0)	43 (86.0)
*GAD-7 Moderate/Severe*	16 (64.0)	15 (60.0)	31 (62.0)
HRQoL (EQ-5D-5L; -0.285 to 1.000)	0.627 (0.244)	0.497 (0.209)	0.562 (0.234)
Comorbidities (medical history)			
* Depression*	6 (24.0)	8 (32.0)	14 (28.0)
*Anxiety*	7 (28.0)	10 (40.0)	17 (34.0)
*Personality disorder*	1 (4.0)	1 (4.0)	2 (4.0)
*Chronic pain condition*	7 (28.0)	6 (24.0)	13 (26.0)
*Fatigue (chronic)*	4 (16.0)	1 (4.0)	5 (10.0)
*Gastrointestinal condition*	5 (20.0)	5 (20.0)	10 (20.0)
*Migraine*	7 (28.0)	5 (20.0)	12 (24.0)
*Epilepsy*	2 (8.0)	1 (4.0)	3 (6.0)
Health care service use in past 6 months			
Number of GP visit or NHS walk-in (med, IQR)	6.0 (1.5,8.5)	3.0 (1.5,8.5)	5.0 (1.8,8.3)
Number of outpatient appointments (med, IQR)	3.5 (2.0,5.0)	2.0 (1.0,3.5)	2.0 (1.0,5.0)
Any diagnostic test administered	18 (72.0)	10 (40.0)	28 (56.0)
NHS 111			
*None*	13 (52.0)	15 (60.0)	28 (56.0)
*1–2*	6 (24.0)	6 (24.0)	12 (24.0)
*≥ 3*	6 (24.0)	4 (16.0)	10 (20.0)
Emergency department visit			
*None*	13 (52.0)	13 (52.0)	26 (52.0)
*1–2*	9 (36.0)	7 (28.0)	16 (32.0)
*≥ 3*	3 (12.0)	5 (20.0)	8 (16.0)
Any call to ambulance	6 (24.0)	11 (44.0)	17 (34.0)
Any night in hospital	4 (16.0)	6 (60.0)	10 (20.0)
Medications prescribed			
*Sleeping tablet*	6 (24.0)	5 (20.0)	11 (22.0)
*Anti-depressant*	14 (56.0)	11 (44.0)	25 (50.0)
*Anti-anxiety*	7 (28.0)	7 (28.0)	14 (28.0)
*Mood stabiliser*	2 (8.0)	1 (4.0)	3 (6.0)
*Anti-psychotic*	0 (0.0)	2 (8.0)	2 (4.0)
*Anti-epileptic medication*	8 (32.0)	2 (8.0)	10 (20.0)

Values are frequencies (percentages) unless otherwise stated

med = median; IQR = inter-quartile range. For the International Trauma Exposure Measure (ITEM), Criterion A (Crit. A) comprises 16 items describing events that are direct or indirect threat to life, or to physical or sexual safety; these reflect the DSM-5 definition of trauma exposure. Criterion B (Crit. B) comprises 5 items describing events that are psychologically (psyc.) threatening and are considered traumatic in line with ICD-11 guidelines; PTSD = Post-Traumatic Stress Disorder; ITQ = International Trauma Questionnaire; PHQ-9 = Patient Health Questionnaire-9 (Moderate/Severe =  ≥ 10); GAD-7 = Generalized Anxiety Disorder-7 (Moderate/Severe =  ≥ 10). HRQoL = Health-Related Quality of Life; EQ-5D-5L = Health State Valuation Score from the EQ-5D-5L measure (range from extreme problems in all domains (-0.285) to no problems in any domain (1.000)). One participant did not have data pertaining to the number of outpatient appointments available

Almost three-quarters (72%) of participants received their FND diagnosis in the same year as or the previous year before trial enrolment, although 74% had also experienced symptoms before this. All but three participants (who noted functional seizures only) chose two functional symptoms for Ecological Momentary Assessment during the trial. Overall, almost two-thirds (64%) indicated experiencing functional seizures, averaging around 10 per week. Four-fifths of participants (80%) reported experiencing functional motor symptoms (e.g., involuntary movements, tremor, weakness) and a third noted experiencing functional cognitive symptoms (for full list, see Table S1). Reported symptoms frequently crossed domains within participants; 22 (44%) participants reported experiencing both functional seizures and functional motor symptoms (EMDR + NPC *N* = 11 (44%); NPC *N* = 11 (44%)), 9 (18%) indicated experiencing both functional motor and cognitive symptoms (EMDR + NPC *N* = 5 (20%); NPC *N* = 4 (16%)) and 7 (14%) reported both functional seizures and functional cognitive symptoms (EMDR + NPC *N* = 4 (16%); NPC *N* = 3 (12%)). FND symptom profiles were closely matched between participants in EMDR + NPC and NPC trial arms.

Twenty (40%) participants met PTSD diagnostic criteria (as determined by diagnostic algorithm of the ITQ), 9 (36%) in the EMDR + NPC condition and 11 (44%) in the NPC condition, most of which presented with complex PTSD. Exposure to both traumatic life and psychologically threatening events across the lifespan was common in trial participants (ITEM) and was comparable between trial arms. Participants in the EMDR + NPC condition tended to report more (types of) events in adolescence than in childhood or adulthood, while those in the NPC condition reported trauma exposure that increased from childhood to adolescence to adulthood (Table S2). There were high rates of moderate–severe depression (88%) and anxiety (62%) for trial participants (comparable between trial arms) as indicated by scores on the PHQ-9 and GAD-7, respectively. About a quarter of participants experienced migraine with a similar proportion reporting a chronic pain syndrome.

Mean EQ-5D-5L health state valuation scores, derived from the norm-based value set developed for England populations indicated poor HRQoL in participants of both trial arms (corresponding EQ-5D-3L norms observed in age-matched healthy UK populations range from 0.93 to 0.78 across ten-year age cohorts from 25–75 years) [62, 78]. Health care service use in the 6 months prior to enrolment varied considerably across trial participants (Table 1); there was a suggestion of a higher number of GP/NHS walk-ins and administration of diagnostic tests for EMDR + NPC participants relative to their NPC counterparts but, conversely, more participants in the NPC condition called ambulance services and stayed overnight in hospital compared to those in the EMDR + NPC condition.

### Protocol violations

There were 4 protocol violations. One participant required 2 sessions (rather than 1) to complete screening and baseline measures, due to technical difficulties. One participant randomised to EMDR initially wished to delay the start of therapy by 6-months but then agreed to continue within the necessary time frame. One participant did not want to start EMDR after randomisation to EMDR + NPC and withdrew from the study. Finally, due to a programming error, two participants (one in each arm) were included with an MDI identity disturbance score of 15 (above clinical cut-off of 14). The clinical decision was made to keep them in the study as they did not have a diagnosis of dissociative identity disorder, and the level of identity disturbance was not thought to be high enough to compromise their engagement with the study. Of note, both these participants’ MDI identity disturbance scores decreased throughout the trial period.

### Intervention and measure completion

All participants enrolled in the study completed baseline measures prior to randomisation (to EMDR + NPC or NPC condition) and were included in ITT analyses. One participant in the EMDR + NPC group withdrew from the study prior to initiation of EMDR and 2 participants did not complete EMDR therapy (one of which withdrew from the study), yielding an intervention adherence rate of 88.0% (CI = 68.8%, 97.5%), above the threshold for feasibility (i.e., 70%).

Ecological momentary assessment (EMA) data were available for 42 of 50 participants at 3-month follow-up (86.0%, CI = 70.9%,92.8%), 40 of 50 at 6-month follow-up (80.0%, CI = 66.3%,90.0%), and 34 of 50 at 9-month follow-up (68.0%, CI = 53.3%,80.5%). Almost 60% of the participants (29 of 50, 58.0%) completed EMA follow-up measures at all time points while data from at least one EMA follow-up were available in 46 of 50 (92.0%) participants. Within each 2-week EMA period, more than three-quarters of participants responded on 5 or more (of the 14) days at baseline (48 of 50, 96.0%) and 3-month (39 of 50, 78.0%) and 6-month (38 of 50, 76.0%) follow-ups indicating a high level of retention for EMA measures. But this rate dropped to below the feasibility criterion of 50% at 9-month follow-up (48.0%; Table S3), although there was no differential retention between EMDR + NPC and NPC conditions. Of those who completed EMA measures on at least one day of the relevant period, overall completion rates were high at baseline (mean number of days EMA completed (M) = 11.4, SD = 2.9) but declined steadily thereafter (3-month FU M = 10.1, SD = 3.6; 6-month FU M = 9.6, SD = 3.4; 9-month FU M = 7.9, SD = 4.7; Table S3).

Standardised questionnaire data were available for 41 of 50 participants at 3-month follow-up (82.0%, CI = 0.69%,0.91%), 42 of 50 at 6-month follow-up (84.0%, CI = 0.71%,0.93%), and 38 of 50 at 9-month follow-up (76.0%, CI = 0.62%,0.87%), meeting the feasibility criteria. Two-thirds of the participants (33 of 50, 66.6%) completed follow-up measures at all time points while data from at least one follow-up were available in 46 of 50 (92.0%,) participants. Retention rates were comparable between conditions at each time period, save for perhaps at 9-month follow-up (EMDR + NPC = 22 of 25, 88.0%; NPC = 17 of 25, 68.0%; Table S3).

### EMDR therapy sessions

The mean number of EMDR sessions completed in the intervention arm of the study was 13.0 (Table S4). The two participants who did not complete their EMDR therapy received 6 and 7 sessions each; all other participants completed a minimum of 8 sessions. Of note, 5 participants were unable to attend 16 sessions in the 6-month period due to running out of time; and 3 participants chose to end therapy before completing 16 sessions as they felt they had improved and did not require more sessions. Sessions ranged from approximately 60 to 80 min, interspersed by a 7–14 day interval. Three-quarters of participants received the intervention in an online form, with a small number (n = 4, 16.7%) receiving a mixture of online and in-person sessions. For almost 80% of participants (19, 79.2%), bilateral tapping was exclusively used during processing. Two-thirds of participants accessed both FND-related memories/images and non-FND distressing or traumatic memories across sessions.

EMDR treatment had, overall, satisfactory protocol adherence according to the threshold of acceptability on the EMDR Fidelity Rating Scale of ≥ 2.0 (range = 0.0–3.0) ​[58]​. Fifteen participants had one of their sessions graded by an independent reviewer, yielding a mean fidelity score of 2.25 (SD = 0.77), with a median score of ≥ 2.0 in all domains on the measure (Re-evaluation, Assessment, Desensitisation, Installation, Body scan, and Closure).

### Interviews

All participants who completed the trial in the EMDR + NPC arm were invited to take part in an interview, and 11 consented to interview*.* Fifteen participants from the NPC arm were invited to interview using purposive sampling, and of these, 7 consented and were interviewed. In total, 18 participants took part in semi-structured interviews between December 2023 and July 2024. The results from qualitative analyses will be presented in separate papers.

### Intervention acceptability and subjective improvement

At their 9-month follow-up, participants in the EMDR + NPC condition evidenced greater belief they had been given the right treatment (g = 0.97, CI = 0.30, 1.64) and higher levels of satisfaction with the study intervention (*g* = 1.18, CI = 0.47,1.83) than those in the NPC condition (Table 3). Eighteen of the 22 (81.8%) participants receiving EMDR stated a preference for this treatment, 2 indicated a preference for NPC only and 2 had no preference. Half of the participants receiving NPC only (8, 50.0%) stated a preference for EMDR + NPC treatment, 3 (18.8%) preferred the trial arm they were randomised to and 5 (31.3%) had no preference for either approach. The CGI data indicated that an overwhelming majority of participants in the EMDR + NPC condition perceived at least some degree of improvement in their FND symptoms and only one participant reported that one of their symptoms was ‘minimally worse’. A good outcome was reported by 77.2% and 57.1% of participants in the intervention condition for FND symptoms 1 and 2, respectively, compared to 18.8% and 6.7% of NPC participants. A similar pattern of ratings was observed in relatives of the participants (Table 2).

**Table 2:** FND care satisfaction and Clinical Global Impression Scale scores at 9-month follow-up

	EMDR + NPC n = 22 Mean (SD)	NPC n = 16 Mean (SD)	Hedge’s g	
Believe given the right treatment (0–10)	8.14 (1.96)	5.50 (3.41)	0.97 (0.30,1.64)	
Satisfied with treatment within the study (0–10)	8.77 (1.46)	6.13 (2.99)	1.18 (0.47,1.83)	

	Self-rating			
	Symptom 1		Symptom 2	
	EMDR + NPC n = 22 Mean (SD)	NPC n = 16 Mean (SD)	EMDR + NPC n = 21	NPC n = 15
Perceived change in condition (1–7)	2.09 (0.75)	3.56 (1.15)	2.48 (0.93)	3.60 (0.74)
	n (%)	n (%)		
Very much improved (1)	4 (18.2)	0 (0.0)	2 (9.5)	0 (0.0)
Much improved (2)	13 (59.1)	3 (18.8)	10 (47.6)	1 (6.7)
Minimally improved (3)	4 (18.2)	5 (31.3)	7 (33.3)	5 (33.3)
No change (4)	1 (4.5)	5 (31.3)	1 (4.8)	8 (53.3)
Minimally worse (5)	0 (0.0)	2 (12.5)	1 (4.8)	1 (6.7)
Much worse (6)	0 (0.0)	1 (6.3)	0 (0.0)	0 (0.0)
Very much worse (7)	0 (0.0)	0 (0.0)	0 (0.0)	0 (0.0)
'Good' outcome	17 (77.2)	3 (18.8)	12 (57.1)	1 (6.7)

	Rating by relative			
	Symptom 1		Symptom 2	
	EMDR + NPC n = 18 Mean (SD)	NPC n = 12 Mean (SD)	EMDR + NPC n = 17 Mean (SD)	NPC n = 12 Mean (SD)
Perceived change in condition (1–7)	2.56 (0.86)	2.56 (0.86)	2.59 (0.87)	3.83 (1.40)
	n (%)	n (%)		
Very much improved (1)	1 (5.6)	1 (8.3)	1 (5.9)	0 (0.0)
Much improved (2)	9 (50.0)	0 (0.0)	7 (41.2)	3 (25.0)
Minimally improved (3)	5 (27.8)	3 (25.0)	8 (47.1)	1 (8.3)
No change (4)	3 (16.7)	5 (41.7)	0 (0.0)	5 (41.7)
Minimally worse (5)	0 (0.0)	1 (8.3)	1 (5.9)	1 (8.3)
Much worse (6)	0 (0.0)	2 (16.7)	0 (0.0)	2 (16.7)
Very much worse (7)	0 (0.0)	0 (0.0)	0 (0.0)	0 (0.0)
'Good' outcome	10 (55.6)	1 (8.3)	8 (47.1)	3 (25.0)

A rating of ‘much improved’ or ‘very much improved’ with respect to FND symptom was considered as a ‘Good' outcome

### Intent-to-treat analyses

**Fig. 2:**
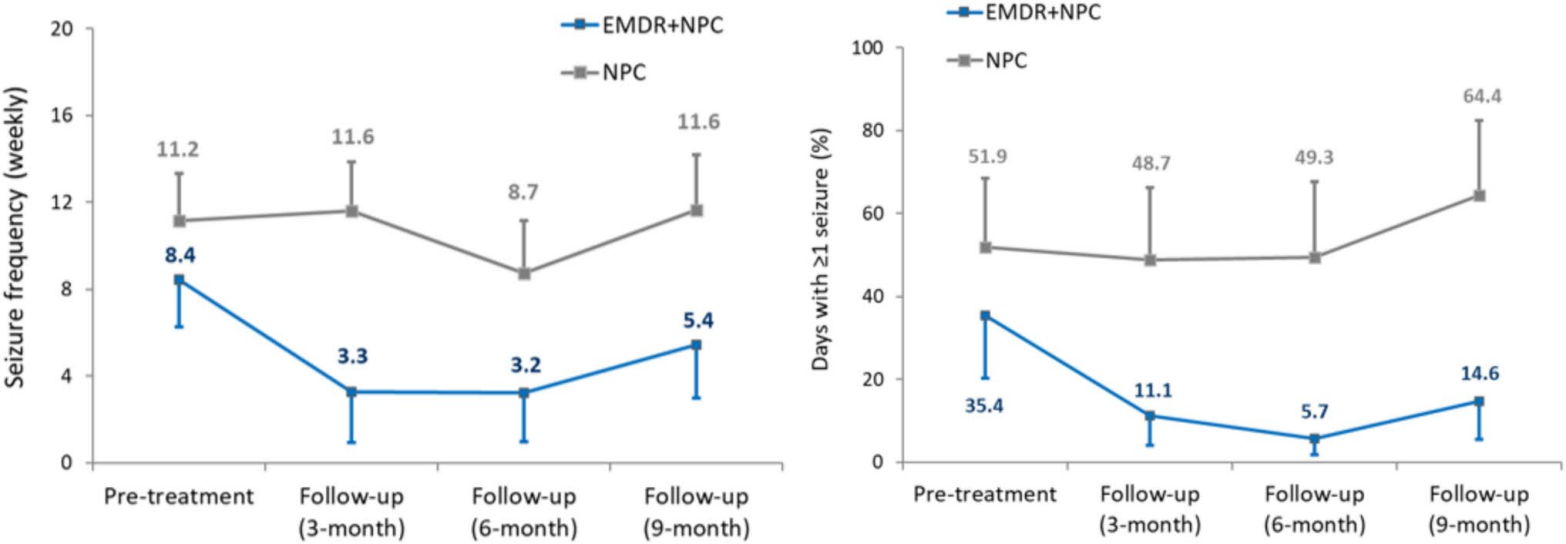
Frequency of seizures per week (**a**) and proportion of days in which one or more seizures were experienced (**b**) by participants in EMDR (+ NPC) and NPC conditions at pre-treatment and follow-up periods (ITT approach). Data labels are adjusted mean frequencies (**a**) and mean percentage values (**b**). Error bars represent standard errors. For seizure frequency, effect sizes were calculated from values that were transformed—raw mean (SE) values are shown for ease of interpretation. At 3-, 6- and 9-month follow, respective between-group Hedge’s *g* (CI) were 0.50 (0.29,0.71), 0.46 (0.21,0.70) and 0.39 (0.14,0.65) for seizure frequency and 0.59 (0.38,0.81), 0.76 (0.51,1.01) and 0.93 (0.66,1.20) for percentage of days with ≥ 1 seizure

**Fig. 3:**
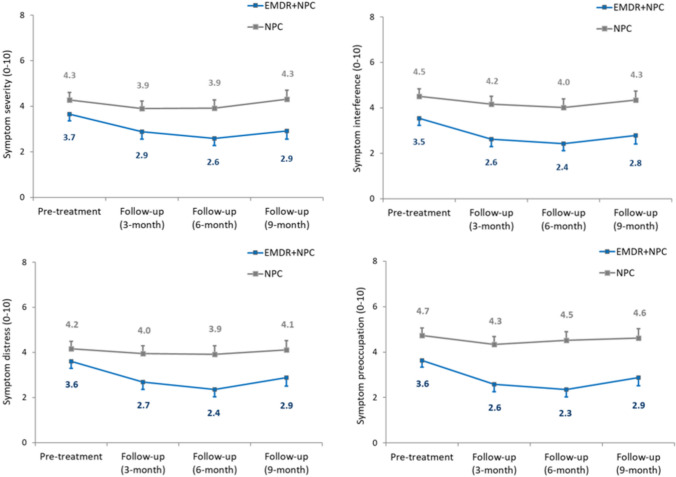
Adjusted mean daily EMA severity (**a**), interference (**b**), distress (**c**) and preoccupation (**d**) ratings for (all) functional symptoms for participants in EMDR (+ NPC) and NPC conditions at pre-treatment and follow-up periods (ITT approach). Data labels are adjusted mean values. Error bars represent standard errors. At 3-, 6- and 9-month follow, respective between-group Hedge’s *g* (CI) were 0.18 (0.06, 0.30), 0.31 (0.17, 0.45) and 0.34 (0.19, 0.49) for Symptom severity, 0.24 (0.12, 0.36), 0.26 (0.13, 0.40) and 0.25 (0.10, 0.39) for Symptom interference, 0.29 (0.17, 0.40), 0.41 (0.27, 0.55) and 0.28 (0.13, 0.42) for Symptom distress, and 0.27 (0.15, 0.38), 0.44 (0.30, 0.58) and 0.26 (0.12, 0.41) for Symptom preoccupation

Results of the ITT analyses of EMA are shown in Figs. 2a-2b and 3a-3d and Table 3. Considering only those participants who experienced functional seizures, both the weekly frequency and proportion of days experiencing a seizure decreased for participants in the EMDR + NPC condition relative to those in the NPC arm (Fig. 2a-2b); between-group effect sizes at each follow-up suggested potential treatment benefits were medium-sized for the former (*g* ranged from 0.39–0.50) and moderate-to-large for the latter (*g* ranged from 0.59 to 0.93). Across all functional symptoms reported by trial participants, there were modest improvements in symptom severity, interference, distress and preoccupation in the EMDR + NPC arm compared with the NPC arm, with between-group effect sizes ranging from 0.18 to 0.44 according to specific measure and time point (Table 3). However, analyses that considered each (broad) symptom type separately (Table 4), suggested little evidence of an advantage of EMDR + NPC treatment for ratings specifically related to functional seizures (*g* ranged across measures and time point from -0.35 to 0.28). In contrast, there were small-to-medium sized between-group effects for ratings of functional motor symptoms (range from 0.06 to 0.52) and (mostly) moderate-to-large effects sizes for ratings of functional cognitive symptoms (range from 0.24 to 0.97; Table 4).

**Table 3:** Adjusted descriptive statistics and between-group effect size estimates for EMA measures of functional symptoms (ITT approach)

	Functional Seizures			Functional Motor Symptoms			Functional Cognitive Symptoms		
	EMDR + NPC	NPC	EMDR + NPC vs NPC	EMDR + NPC	NPC	EMDR + NPC vs NPC	EMDR + NPC	NPC	EMDR + NPC vs NPC
	Mean (SE)	Mean (SE)	Hedge’s g (95% CI)	Mean (SE)	Mean (SE)	Hedge’s g (95% CI)	Mean (SE)	Mean (SE)	Hedge’s g (95% CI)
Severity									
Baseline	2.46 (0.39)	3.16 (0.42)		3.86 (0.34)	5.15 (0.35)		4.65 (0.49)	4.54 (0.57)	
3-Month F-U	1.45 (0.43)	2.71 (0.44)	0.28 (0.07,0.48)	3.26 (0.38)	4.71 (0.36)	0.06 (-0.10,0.23)	3.91 (0.54)	4.30 (0.60)	0.24 (-0.06,0.54)
6-Month F-U	1.47 (0.41)	2.13 (0.47)	-0.02 (-0.27,0.22)	2.57 (0.37)	4.75 (0.39)	0.37 (0.18,0.56)	3.71 (0.52)	4.86 (0.69)	0.60 (0.24,0.97)
9-Month F-U	1.91 (0.45)	2.27 (0.49)	-0.17 (-0.43,0.08)	2.96 (0.41)	4.90 (0.41)	0.27 (0.07,0.47)	3.89 (0.64)	5.77 (0.77)	0.96 (0.53,1.39)
Interference									
Baseline	2.39 (0.40)	3.81 (0.43)		3.61 (0.35)	5.06 (0.36)		4.61 (0.50)	4.67 (0.58)	
3-Month F-U	1.34 (0.44)	3.21 (0.45)	0.20 (-0.01,0.41)	2.78 (0.39)	4.77 (0.37)	0.22 (0.06,0.39)	3.75 (0.55)	4.51 (0.61)	0.35 (0.05,0.66)
6-Month F-U	1.38 (0.42)	2.61 (0.47)	-0.09 (-0.34,0.15)	2.18 (0.37)	4.89 (0.40)	0.52 (0.33,0.72)	3.72 (0.53)	4.55 (0.70)	0.39 (0.03,0.74)
9-Month F-U	1.83 (0.46)	2.71 (0.50)	-0.25 (-0.51,0.01)	2.61 (0.41)	4.90 (0.42)	0.35 (0.15,0.55)	3.92 (0.64)	5.39 (0.77)	0.71 (0.29,1.13)
Distress									
Baseline	2.84 (0.40)	4.19 (0.42)		3.60 (0.35)	4.60 (0.35)		4.38 (0.49)	3.68 (0.58)	
3-Month F-U	1.94 (0.44)	3.40 (0.44)	0.04 (-0.16,0.25)	2.87 (0.39)	4.53 (0.37)	0.25 (0.08,0.41)	3.26 (0.54)	3.92 (0.61)	0.60 (0.29,0.91)
6-Month F-U	1.59 (0.42)	2.92 (0.47)	-0.01 (-0.26,0.23)	2.13 (0.37)	4.43 (0.40)	0.49 (0.30,0.68)	3.33 (0.53)	4.42 (0.91)	0.79 (0.42,1.16)
9-Month F-U	2.13 (0.46)	2.85 (0.50)	-0.27 (-0.52,-0.01)	2.60 (0.41)	4.59 (0.42)	0.37 (0.18,0.57)	3.90 (0.64)	4.92 (0.77)	0.75 (0.33,1.18)
Preoccupation									
Baseline	3.07 (0.40)	5.48 (0.43)		3.53 (0.35)	4.86 (0.36)		4.32 (0.50)	3.86 (0.58)	
3-Month F-U	1.85 (0.44)	4.17 (0.45)	-0.03 (-0.24,0.18)	2.71 (0.39)	4.62 (0.37)	0.22 (0.05,0.38)	3.20 (0.55)	4.23 (0.61)	0.67 (0.36,0.98)
6-Month F-U	1.68 (0.42)	4.19 (0.47)	0.04 (-0.20,0.29)	2.03 (0.37)	4.70 (0.40)	0.50 (0.30,0.69)	3.34 (0.53)	4.69 (0.70)	0.82 (0.45,1.19)
9-Month F-U	2.26 (0.46)	3.79 (0.50)	-0.35 (-0.61,0.09)	2.84 (0.41)	4.84 (0.42)	0.25 (0.05,0.45)	3.54 (0.65)	5.23 (0.78)	0.97 (0.54,1.40)

Three participants did not indicate a second FND symptom and, as such, did not provide any VAS ratings. Effect sizes = Hedge’s g; Positive effect sizes represent an improvement in symptoms that is greater in EMDR + NPC relative to NPC

**Table 4:** Adjusted descriptive statistics and between-group effect size estimates for ITQ subscales and measures of HRQoL and disability (ITT approach)

	EMDR + NPC Mean (SE)	NPC Mean (SE)	EMDR + NPC vs NPC Effect size (95% CI)
PTSD			
ITQ DSO Symptom Score (0–24)			
Baseline	12.64 (1.20)	12.20 (1.20)	
3-month Follow-up	10.74 (1.29)	11.84 (1.26)	0.24 (-0.31,0.79)
6-month Follow-up	8.69 (1.25)	12.00 (1.27)	0.59 (0.03,1.15)
9-month Follow-up	9.09 (1.26)	10.31 (1.39)	0.27 (-0.29,0.81)
ITQ CPTSD Symptom Score (0–48)			
Baseline	24.28 (2.19)	24.76 (2.19)	
3-month Follow-up	18.67 (2.33)	24.42 (2.28)	0.45 (-0.10,1.00)
6-month Follow-up	16.01 (2.27)	23.31 (2.31)	0.58 (0.03,1.14)
9-month Follow-up	16.20 (2.28)	22.65 (2.51)	0.51 (-0.04,1.07)
HRQoL/Disability			
WHODAS 2.0 Total (0–100)			
Baseline	43.83 (3.93)	53.39 (3.93)	
3-month Follow-up	39.20 (4.11)	52.04 (4.03)	0.16 (-0.39,0.71)
6-month Follow-up	35.26 (4.02)	52.65 (4.06)	0.38 (-0.17,0.93)
9-month Follow-up	37.42 (4.04)	50.53 (4.29)	0.17 (-0.37,0.72)
EQ-5D-5L Value (0–100)			
Baseline	0.627 (0.051)	0.497 (0.051)	
3-Month follow-up	0.675 (0.053)	0.477 (0.052)	0.32 (-0.23,0.87)
6-Month ollow-up	0.646 (0.052)	0.485 (0.052)	0.18 (-0.37,0.72)
9-Month follow-up	0.613 (0.052)	0.547 (0.056)	-0.23 (-0.77,0.32)
EQ-VAS (0–100)			
Baseline	50.24 (3.87)	44.72 (3.87)	
3-Month follow-up	55.19 (4.36)	41.71 (4.13)	0.42 (-0.13,0.98)
6-Month follow-up	59.09 (4.09)	42.19 (4.19)	0.61 (0.05,1.16)
9-Month follow-up	51.52 (4.11)	52.25 (4.72)	-0.34 (-0.88,0.22)

MDI = Multiscale Dissociation Inventory; PTSD = Post-Traumatic Stress Disorder; ITQ = International Trauma Questionnaire; DSO = Disturbances in Self-Organization; CPTSD = Complex Post-Traumatic Stress Disorder; HRQoL = Health-related quality of life; WHODAS 2.0 = WHO Disability Assessment Schedule version 2.0. WHODAS 2.0 mean (SD) values were calculated using complex scoring, based on item response theory-based scoring (which accounts for multiple levels of difficulty for each WHODAS item; Üstün, 2010): EQ-5D-5L = EQ-5D-5L health state evaluation EQ-VAS = current overall health rating (today); PHQ-9 = Patient Health Questionnaire-9; GAD-7 = Generalized Anxiety Disorder-7. Effect sizes = Hedge’s g; Positive effect sizes represent an improvement in symptoms that is greater in EMDR + NPC relative to NPC. For EQ-5D-5L, effect sizes were calculated from values that were transformed – raw mean (SE) values are shown for ease of interpretation

**Fig. 4:**
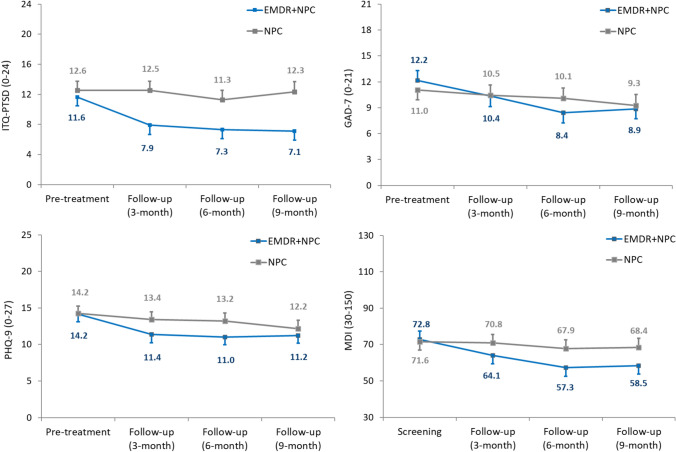
International Trauma Questionnaire (ITQ)-PTSD (**a**), Generalized Anxiety Disorder-7 (GAD-7; **b**), Patient Health Questionnaire-9 (PHQ-9; **c**) and Multiscale Dissociation Inventory scores (MDI total; **d**) for participants in EMDR (+ NPC) and NPC conditions at screening and follow-up periods (ITT approach). Data labels are adjusted mean values. Error bars represent standard errors. At 3-, 6- and 9-month follow, respective between-group Hedge’s *g* (CI) were 0.60 (0.04,1.15), 0.49 (-0.06,1.05) and 0.71 (0.13,1.26) for ITQ-PTSD, 0.23 (-0.32,0.78), 0.52 (-0.08,1.12) and 0.28 (-0.31,0.87) for GAD-7, 0.41 (-0.14,0.96), 0.44 (-0.15,1.03) and 0.18 (-0.41,0.76) for PHQ-9 and 0.33 (-0.22,0.88), 0.49 (-0.07,1.04) and 0.46 (-0.09,1.01) for MDI total

Exploratory analyses considering (ITQ-derived) PTSD status at baseline suggested more pronounced EMDR-associated decreases in seizure frequency reductions in individuals not meeting criteria for PTSD at baseline (g ranged from 0.62 to 0.71) than those meeting criteria (g ranged from 0.16 to 0.36; see Table S5). Treatment benefits across symptom ratings (all types) tended to be smaller at 3-month follow-up for those meeting criteria for PTSD at baseline (relative to those not meeting criteria) but larger at later follow-ups (see Table S5),

Participants receiving EMDR + NPC reported decreased levels of PTSD symptoms, dissociation, depression and anxiety over the course of the study (Fig. 4a-4d). The most consistent signal of efficacy for EMDR + NPC was for PTSD symptoms, where between-group effect sizes ranged from 0.49 to 0.71 at follow-ups; the degree of treatment benefit was largely comparable between those meeting criteria for PTSD at baseline (*g* ranged from 0.47 to 0.90) and those not meeting criteria (*g* ranged from 0.43 to 0.76; see Figure S1a), although early improvements were more pronounced for individuals who experienced functional seizures (see Table S6). ITQ subscale analyses suggested the advantage of EMDR + NPC was more consistent over time for symptoms relating to complex PTSD (0.45 to 0.58) rather than Disturbances in Self-Organization (*g* = 0.24 to 0.59; Table 4). Treatment benefits tended to be more modest for measures of mood and dissociation, in part due to a small improvement on each for participants in the NPC condition (particularly at 9-month follow-up); nevertheless, effect sizes at 6-month follow-up for GAD-7 (*g* = 0.52, CI = -0.08,1.12), PHQ-9 (*g* = 0.44, CI = -0.15,1.03) and MDI total (*g* = 0.49, CI = -0.07,1.04) suggested effects that were moderate (or close to moderate) in magnitude. Across measures of HRQoL and functional disability, findings were less consistent. At 3- and 6-month follow-ups participants receiving EMDR + NPC reported improved overall health on the EQ-VAS (mean increases of between 5.0 and 8.9 points, respectively), yielding moderate effect sizes (0.42 to 0.61), but differences with participants in the NPC arm were diminished at 9 months. A similar pattern emerged with changes in EQ-5D-5L health state evaluation scores and (decreased) disability on the WHODAS 2.0, although the early advantage in the EMDR + NPC arm was more modest (Table 4).

### Per-protocol analyses

The pattern of findings considering only data at the relevant time points, and, in the active intervention condition, those participants who completed EMDR treatment (‘per-protocol’) was similar to that yielded from ITT analyses, particularly for EMA measures, where between-group effect sizes for seizure frequency and ratings at each time point were closely aligned (see Supplementary Material; Tables S7-S9). Differences between per-protocol participants in the EMDR + NPC and NPC conditions for ITQ-PTSD symptoms were moderate-to-large across follow-ups (0.59 to 0.75) at 3- and 6-month follow-ups, while between-group effect sizes at 3- and 6-month follow-ups for measures of anxiety (0.30, 0.51), depression (0.61, 0.49), disability (0.33, 0.57) and HRQoL (EQ-5D-5L health state evaluation 0.58, 0.30; EQ-VAS 0.50, 0.77) were slightly elevated in comparison with those observed in ITT analyses (see Supplementary Material; Table S10).

### Adverse events

Thirteen participants in the EMDR + NPC condition and 6 participants in the NPC condition reported one or more adverse events/reactions over the course of the study.

Six participants experienced an adverse reaction to EMDR treatment and/or trial participation: 3 reported an increase in FND symptoms (functional seizures or dissociative episodes) following the beginning of EMDR sessions, one of which required hospitalisation (serious adverse reaction), while 3 reported other trial-related phenomena (suicidal ideation, mild injury sustained during session, and emotional triggering when completing questionnaires).

In the EMDR + NPC arm, no participant reported a serious adverse event. However, 7 participants reported one or more adverse events; 3 individuals experienced an adverse event directly linked to (pre-existing) FND symptoms (relapse in symptoms, injury sustained as a result of functional seizures), 2 reported a deterioration in mental health (one of these led to self-harm episode), 3 experienced adverse events that were attributable to a COVID-19 or influenza infection, 2 reported migraines (one of which attended ED), and another reported relapse of fibromyalgia and Crohn’s disease.

In the NPC trial arm, 4 participants reported a serious adverse event which required hospitalisation and/or surgery (2 of these were associated with (pre-existing) FND symptoms). Six participants (including 2 that also reported a serious adverse event) reported an adverse event (injury sustained as a result of functional seizure, self-harm and suicidal ideation, infection, sleep difficulties, diagnosis or procedure relating to non-FND illnesses).

### Health care utilisation

Data pertaining to neuropsychiatry service use during the trial period, available for all but two participants in the EMDR + NPC condition (both did not complete EMDR therapy), indicated that most participants in each trial arm had one or more appointments with a neuropsychiatry consultant (EMDR + NPC: 1 appointment n = 6, 26.1%; 2–3 appointments n = 13, 56.5%; NPC: 1 appointment n = 11, 44.0%; 2–3 appointments n = 13, 50.0%). Around a third of participants had attended a FND education session (EMDR + NPC n = 8, 34.8%; NPC n = 7, 28.0%) while a fifth had participated in a functional seizure group (EMDR + NPC n = 4, 17.4%; NPC n = 6, 24.0%). Almost half of EMDR + NPC participants (n = 11, 47.8%) were discharged from neuropsychiatry services by trial end compared with a quarter of NPC participants (n = 6, 24,0%).

Summary data describing the previous 6 months of health care resource use at baseline and (subsequent) 9 months of health care resource use at 9-month follow-up (for those with data at both time points) are shown in Table 5. Primary care utilisation, use of NHS 111 (non-emergency healthcare telephone line) and the number of outpatient appointments decreased over the course of the study for participants receiving EMDR + NPC, while they remained constant or increased for participants in the NPC arm. The proportion of participants in the EMDR + NPC condition visiting the emergency department, making one or more calls to ambulance services and being hospitalised were largely unchanged although at 9-month follow-up remained lower than their counterparts in the NPC condition. Use of sleeping tablets and anti-anxiety, mood stabiliser, and/or anti-epileptic medication were generally low at both time points for trial participants. Nevertheless, the number of participants prescribed sleeping tablets decreased from baseline to 9-month follow-up for participants receiving EMDR + NPC but increased for NPC participants, and 3 participants in the NPC condition had initiated use of mood stabilisers at 9-month follow-up.

**Table 5:** Healthcare utilisation in past 6 months at baseline and in the past 9 months at 9-month follow-up for FND participants in EMDR + NPC (n = 22) and NPC (n = 16) intervention conditions

	EMDR+NPC		NPC	
	Baseline	9-Month FU	Baseline	9-Month FU
Number of GP visit or NHS walk-in (med, IQR)	5.5 (1.0,8.3)	2.0 (1.0,5.3)	3.0 (2.0,10.0)	4.5 (2.0,9.0)
Number of outpatient appointments (med, IQR)	3.0 (1.5, 5.0)	1.0 (0.0,3.5)	2.0 (1.0,4.5)	2.0 (1.0,3.0)
NHS 111				
*None*	12 (54.5)	15 (68.2)	10 (62.5)	8 (50.0)
*1–2*	6 (27.3)	6 (27.3)	3 (18.7)	5 (31.3)
*≥ 3*	4 (18.2)	1 (4.5)	3 (18.7)	3 (18.7)
Emergency department visit				
*None*	12 (54.8)	13 (59.1)	8 (50.0)	5 (31.3)
*1–2*	8 (36.4)	8 (36.4)	4 (25.0)	9 (56.3)
*≥ 3*	2 (9.1)	1 (4.5)	4 (25.0)	2 (12.5)
Any call to ambulance	4 (18.2)	5 (22.7)	7 (43.8)	6 (37.5)
Any diagnostic test	16 (72.7)	6 (27.3)	7 (42.9)	6 (37.5)
Any night in hospital	3 (13.6)	3 (13.6)	5 (31.3)	5 (31.3)
Medications prescribed				
*Sleeping tablet*	5 (22.7)	1 (4.5)	2 (12.5)	5 (31.3)
*Anti-anxiety medication*	6 (27.3)	7 (31.8)	5 (31.3)	4 (25.0)
*Mood stabiliser*	2 (9.1)	1 (4.5)	0 (0.0)	3 (18.8)
*Anti-epileptic medication*	7 (31.8)	5 (22.7)	2 (12.5)	3 (18.8)

Values represent frequencies (percentages) unless otherwise stated

Data include only those participants completing measures at both baseline and 9-month follow-up. med = median; IQR = interquartile range

## Discussion

This randomised controlled feasibility study met the pre-defined criteria, by demonstrating an adequate recruitment rate (58% of those referred became participants, and they were recruited within the 1-year target recruitment time period); good intervention adherence (88% randomised to EMDR + NPC completed therapy); and good retention in the trial (76% participants completed outcome measures at final measurement point). Although the recruitment rate did not reach the 70% target, the recruitment rate is similar to the large CODES study of CBT for functional seizures (698 participants originally consented to an observation period with 368 participants ultimately consented and randomised: 53%), and as such our percentage target was perhaps too ambitious and could be lower in a future trial [24, 29]. Retention rates were similar across arms, although with less completion for those in the NPC arm at 9-month follow-up (68% completion vs. 88% completion in EMDR + NPC arm). Therefore, this study has demonstrated feasibility in terms of recruitment, retention, and treatment adherence.

Across both arms, there were no unexpected adverse events or adverse reactions. Six participants in the EMDR + NPC experienced adverse reactions (e.g. increase in FND symptoms, increase in suicidal ideation), with one serious adverse reaction as one participant admitted to hospital due to the severity of their FND symptoms (which had happened to them previously). Similar levels of adverse events occurred across both arms, but there were no serious adverse events in the EMDR + NPC arms, whereas there were 4 serious adverse events in the NPC arm (participants required hospitalisation, with 2 admissions due to worsening of FND). The nature of the adverse events/reactions are expected in this patient population irrespective of trial participation. Participants receiving EMDR were warned that they might experience an increase in their physical symptoms or distress, or worsening of mental health difficulties during therapy, and these are known possible effects [34]. We believe that this study shows that EMDR can be delivered safely to people with FND by qualified practitioners accessing EMDR clinical supervision, as long as adequate screening for suitability for EMDR therapy takes place. We excluded any potential participant who was actively suicidal, psychotic, currently alcohol and/or illegal substance dependent, or with an eating disorder. We also excluded any potential participant with a diagnosis of dissociative identity disorder and screened for clinical levels of identity dissociation using the MDI. The EMDR therapy also included careful consideration regarding which memories to focus on, dependent on participants’ personal history and formulation. The trial therapists had completed their EMDR training, but were not yet accredited EMDR therapists, and as such cannot be considered EMDR specialists. The therapy delivered within the trial is therefore likely a good representation of therapy delivered within a national health service where therapists are unlikely to be specialist providers; although the trial therapists did have access to EMDR supervision delivered by an experienced EMDR Consultant. Independent ratings of randomly selected EMDR processing sessions achieved mean adequate fidelity ratings.

FND and trauma have a complicated history, and there has been considerable effort to move away from the simplistic idea that experiencing a traumatic event (particularly sexual abuse) causes FND, and to generate greater understanding of how a complex brain-body disorder like FND develops [79]. The EMDR therapy delivered in the EMDR + NPC arm in this study followed an FND-specific protocol (though still in accordance with EMDR standard protocol) and focused particularly on any distressing memories associated with FND, and therapy therefore did not necessarily address any earlier traumatic memories (even if present). Most participants chose to focus on both FND-specific and non-FND distressing memories within their EMDR, with only 5 participants choosing to focus solely on distressing memories from earlier life. Participants choosing to focus on FND-specific memories, such as their onset of FND symptoms or treatment within the healthcare system in relation to their FND, suggests that experiencing FND itself (and consequences) can be experienced as traumatic, and that it may not be necessary to focus on any non-FND distressing memories from earlier life (if present). The therapy delivered also included FND education that acknowledged possible associations between developing FND and past traumatic experiences, but did not suggest that past traumatic experiences caused their FND. Participants were also educated regarding the roles attention and predictive processing play in the production of FND symptoms.

Baseline PTSD rates, calculated by responses on the ITQ, suggested 40% met criteria for PTSD, with 75% of those also meeting criteria for Complex PTSD (CPTSD). This is much higher than prevalence rates reported in a United States population-based study, where 7.2% of the sample met criteria for either PTSD or CPTSD on the ITQ, with prevalence rates of 3.4% and 3.8%, respectively [80]. The ITQ is a self-report questionnaire in line with ICD-11 diagnostic criteria for PTSD/CPTSD. A key way the ICD-11 differs from DSM-5 criteria is that the DSM-5 requires a “Criterion A” traumatic event to be experienced (direct or indirect exposure to actual or threatened death or serious injury, or learning about such events; and also includes sexual violence, and repeated exposure indirectly or directly through work-related activities) [81]. The ICD-11 only provides guidance regarding what would be classified as a traumatic event, however, namely that an event is experienced as extremely threatening or horrific [82]. Psychologically threatening events (“Criterion B” events), such as bullying, emotional abuse or neglect, and stalking can also result in PTSD symptomatology. There has been concern that not having an explicit requirement for a “Criterion A” traumatic event would lead to inflated PTSD rates, but this has not been found to be the case [63], and it has been found to measure reliable and clinically significant change after treatment [83]. Our sample included participants with relatively high exposure to traumatic events across the lifetime (mean traumatic events = 8.4 events; Mean Criterion A events 4.1, Mean Criterion B events 4.3). All participants had to endorse they had experienced at least one traumatic event on the ITEM for inclusion in the study, and no potential participant was excluded on this basis. Higher rates of traumatic events have been found in the FND population, when compared to healthy controls, but not all patients with FND identify historical traumatic events [10], and the definition of “traumatic” likely plays a part here. The ITEM measures both Criterion A (items 1–16) and Criterion B traumatic events (items 17–22), and as such can be considered to be a broad measure of lifetime exposure to traumatic events. According to a population-based study carried out by the World Health Organisation that included 24 countries, 70.4% of respondents had experienced at least one Criterion A traumatic event, and 52.3% had experienced two or more. The mean number was 3.2 in the WHO study, which indicates our sample experienced a greater number of Criterion A traumatic events than would be expected [84]. This may explain the relatively higher rates of PTSD, but the PTSD rates are similar to those reported in other FND studies [42, 85]. PTSD is more prevalent in those with other psychiatric conditions [86], and the elevated rates in PTSD in our sample could be associated with elevated anxiety and depression reported by our participants (88% and 62% of the sample scored in the Moderate/Severe ranges on the PHQ-9 and GAD-7, indicating depression and anxiety were common co-morbidities). Additionally, it is established that greater exposure to traumatic events, particularly in childhood, is associated with poorer mental and physical health generally [38]. Dissociative symptoms and disorders are associated with more severe FND symptoms and worse quality of life [87]. Mean scores of dissociation, as measured by the MDI, were in the clinical ranges for the following sub-scales: Disengagement, Depersonalisation, Derealisation, and Memory Disturbance; and in the sub-clinical ranges on Emotional Constriction and Identity Disturbance, in line with other studies [88, 89].

Examining the chosen functional neurological symptoms to rate on the Ecological Momentary Assessment (participants chose a maximum of 2 FND symptoms), suggests the participant sample commonly had more than one FND symptom with 47/50 participants choosing to rate 2 symptoms. The most common FND symptoms were functional motor symptoms (80%), followed by functional seizures (64%) with a mean frequency of 9.8 functional seizures per week, and 32% choosing functional cognitive symptoms. 44% participants experienced both functional seizures and functional motor symptoms. The baseline characteristics indicate that our sample is representative of the heterogeneity and complexity of FND presentations [13].

Participants in EMDR + NPC, compared to NPC, reported higher satisfaction with their treatment in the study; as well as greater belief they were given the right treatment. Participants in EMDR + NPC also reported a “good” outcome (much improved or very much improved on CGI) at 9-month follow-up at much higher rates compared to the NPC group (77% vs. 19% for Symptom 1; 57% vs. 7% for Symptom 2).

This was a feasibility study and therefore did not include a large enough sample to test for efficacy. However, comparing EMA data and outcome measures across the 2 arms generally mirrored the participant improvement ratings, and suggests potential benefit of EMDR for FND. In terms of functional seizures, participants in EMDR + NPC experienced fewer functional seizures at the end of the trial (mean 5.4 vs 11.6) and on fewer days (mean 14.6% vs 64.4%), compared to NPC, with moderate-large effect sizes found. A recent meta-analysis reported a moderate pooled effect size (d = 0.53) in terms of seizure improvement across 11 psychological therapy studies, suggesting our outcomes in terms of functional seizures are similar to outcomes in previous studies [30]. Interestingly, this reduction in functional seizures was not reflected in EMDR + NPC participants’ Ecological Momentary Assessment ratings of severity, interference, distress and preoccupation in reference to functional seizures, with the EMDR + NPC and NPC groups showing similar rate of reductions across the trial period. This may be due to higher baseline ratings of NPC participants, but also could be reflective of the service setting, which sees a large number of patients with functional seizures and there is an established functional seizure treatment pathway in the service (patients are invited to an online functional seizure group). Conversely, participants’ rating of severity, interference, distress and preoccupation in reference to functional motor symptoms and cognitive symptoms were lower in EMDR + NPC compared to NPC (small-moderate effect sizes for motor symptoms, and moderate-large effect sizes for cognitive symptoms). Examining Ecological Momentary Assessment ratings of functional symptoms all together showed small-moderate between group effect sizes, and similar effect sizes were found in those categorised as with or without PTSD (at start of study). More than 75% of participants completed ratings on 5 or more (out of 14 days) at baseline, and 3 and 6 month follow-ups, but only 48% of participants completed ratings on 5 or more days at 9 month follow-up. This suggests Ecological Momentary Assessment can be a useful and acceptable tool for measuring FND symptoms and their impact, but that participants may struggle to continue to give regular ratings over a longer time period.

Signals of efficacy were also found on outcome measures, with lower levels of PTSD symptoms, dissociation, depression, and anxiety reported at the end of the trial for EMDR + NPC, in particular for PTSD. Improvements in PTSD symptoms in EMDR + NPC were found for both participants categorised with or without PTSD at the start of the study; and for those with and without functional seizures. These results suggest that FND-focused EMDR may be of benefit to those with or without PTSD, and for a variety of FND presentations.

On a measure of disability (WHODAS), participants reported less disability in EMDR + NPC at the end of the trial (small between groups effect size at 9-months), but in terms of HRQoL (measured by EQ-5D-5L) the ratings were less consistent, with a small between group effect size at 3 and 6 months, which almost disappeared at 9 months. Ratings on the EQ-VAS were improved in the EMDR + NPC group at 6 months (moderate effect size), but this also disappeared at 9 months. These results are surprising given participants subjective ratings of improvement were higher in the EMDR + NPC group and suggest that the measures chosen may not have fully captured changes in functioning and perceived HRQoL. Results in terms of healthcare utilisation were more consistent, with participants in EMDR + NPC using less healthcare resources (GP visits, outpatient appointments, sleeping tablet prescriptions) over the course of the trial, whereas the level of use did not change or increased for NPC participants. Interestingly, using less healthcare resources in the EMDR + NPC arm did not extend to being less likely to access emergency services (i.e. emergency department visit or calling an ambulance), and this is difficult to interpret without knowing reasons for use of emergency services (we did not record whether the use was due to FND or other health conditions).

This feasibility study was a pragmatic study carried out in a specialist national health setting in the UK. Offering EMDR sessions in-person or remotely allowed greater flexibility in delivery, and most participants chose to attend sessions online. A limitation of the study is that there were certain elements that could not be controlled for, such as the number of neuropsychiatrist appointments in the trial period or whether participants attended the FND educational groups offered by the service. It was also not possible for participants to be blind to treatment allocation, and the nature of the trial arms meant that participants had more clinician-contact overall in the EMDR + NPC arm compared to the NPC arm. It was challenging for some participants in the EMDR + NPC arm to schedule 16 sessions in the required 6-month period. The number of sessions offered and/or time frame may need to be adjusted in a future trial. Other trial limitations include that more than a third of potential participants declined further involvement or did not respond to research team contact, but we did not record their characteristics, so do not know whether there were any unifying characteristics of this group. Additionally, we did not carry out a standardised clinical interview to establish PTSD diagnosis presence, rather we classified PTSD status based on ITQ-completion. Finally, there were baseline differences on EMA ratings given for FND symptoms which made direct comparisons of change over time complicated.

In summary, this randomised feasibility study demonstrated feasibility and acceptability, as well as promising results in terms of FND symptom improvement, better mental health, reduced disability, and reduced healthcare costs for those who attended EMDR therapy based on an FND-specific protocol. A full-scale trial is required to establish efficacy of FND-focused EMDR therapy for FND. Our results suggest that using subjective ratings, like the CGI or Ecological Momentary Assessment may be more meaningful markers of improvement for FND patients, in the absence of there being a validated questionnaire that assesses change in FND symptoms. However, the choice of a primary outcome measure in a full-scale trial has not been clearly established.

## Supplementary Information

Below is the link to the electronic supplementary material.

Supplementary file1 (PDF 400 kb)

## Data Availability

Pseudoanonymised participant data is available upon reasonable request from the corresponding author Dr Sarah Cope (sarah.cope@swlstg.nhs.uk). Conditions for reuse will need to be discussed with the corresponding author on a case-by-case basis.
