# Supplementary material for: Randomised feasibility study evaluating eye movement desensitisation and reprocessing therapy for functional neurological disorder (MODIFI)
Source: J Neurol. 2025 Jul 8;272(8):493. doi: 10.1007/s00415-025-13219-5 (PMC12238153; doi:10.1007/s00415-025-13219-5)
Supplement: Supplementary file 1 — Supplementary file1 (PDF 400 kb) [file 415_2025_13219_MOESM1_ESM.pdf]

Supplementary Materials

Article: Randomised feasibility study evaluating eye movement desensitisation and reprocessing therapy for functional neurological disorder (MODIFI)

By Sarah R. Cope[1,2,3], Jared G. Smith[1,4], Sharif El-Leithy[5], Serena Vanzan[1], Patricia Hogwood, Dawn Golder, Kati Jane Turner, Maeve Crowley[6], Jo Billings[7], Susannah Pick[8], Caitlin Pentland[8], Mark J. Edwards[9]

[1]Clinical Research Unit, South West London & St George's Mental Health Trust, Springfield University Hospital, 61 Glenburnie Road, London, SW17 7DJ, UK

[2] Department for Clinical Neuropsychology and Clinical Health Psychology, St George's University Hospitals Foundation NHS Trust, Blackshaw Road, London, SW17 0QT

[3] Inpatient Neuropsychiatry Services, South London & Maudsley NHS Foundation Trust, Maudsley Hospital, Denmark Hill, London, SE5 8AZ, UK

[4]Population Health Research Institute, St George's, University of London, Cranmer Terrace, Tooting, London, SW17 0RE, UK

[5]Traumatic Stress Service, South West London & St. George's Mental Health NHS Trust, Springfield University Hospital, 61 Glenburnie Rd, London SW17 7DJ, UK

[6]Sussex Partnership NHS Trust, Portland House, Richmond Road, Worthing, West Sussex, BN11 1HS, UK

[7]Department of Psychiatry, University College London, Wing A, 4th floor, Maple House, 149 Tottenham Court Road, London W1T 7NF, UK

[8]Department of Psychological Medicine, Institute of Psychiatry, Psychology & Neuroscience, King's College London, London, SE5 8AF, UK

[9]Department of Basic and Clinical Neuroscience, Institute of Psychiatry, Psychology & Neuroscience, King's College London, London, SE5 8AF, UK

Corresponding author: sarah.cope@swlstg.nhs.uk

Journal of Neurology

Table S1. Functional symptom characteristics of participants in EMDR (+NPC; n=25) and NPC (n=25) conditions.

	EMDR+NPC	NPC	Total
	n (%)	n (%)	n (%)
Functional symptom 1	n=25	n=25	n=50
Functional/Dissociative seizures	13 (52.0)	16 (64.0)	29 (58.0)
Motor symptoms			
Involuntary twitches/movements	6 (24.0)	2 (8.0)	8 (16.0)
Tremor	3 (12.0)	3 (12.0)	6 (12.0)
Weakness	0 (0.0)	2 (8.0)	1 (2.0)
Gait disturbance	0 (0.0)	1 (4.0)	1 (2.0)
Visual problems	1 (4.0)	0 (0.0)	1 (2.0)
Cognitive symptoms	2 (8.0)	1 (4.0)	2 (4.0)
Functional symptom 2	n=24	n=23	n=47
Functional/Dissociative seizures	3 (12.5)	0 (0.0)	3 (6.4)
Motor symptoms			
Involuntary twitches/movements	4 (16.7)	4 (17.4)	8 (17.0)
Tremor	3 (12.5)	0 (0.0)	3 (6.4)
Weakness	2 (8.3)	4 (17.4)	6 (12.8)
Gait disturbance	0 (0.0)	2 (8.7)	2 (4.3)
Visual problems	1 (4.2)	2 (8.7)	3 (6.4)
Speech difficulties	1 (4.2)	1 (4.3)	2 (4.3)
Other	3 (12.5)	4 (17.4)	7 (14.9)
Cognitive symptoms	7 (29.2)	6 (26.1)	13 (27.7)

Note: ‘Other’ (functional) motor symptoms include paralysis, limb numbness, dystonia, dizziness and sensory symptoms.

Table S2. Exposure to traumatic events at screening for participants in EMDR (+NPC; n = 25) and NPC (n = 25) conditions.

	EMDR+NPC	NPC	Total
	Mean (SD;range)	Mean (SD;range)	Mean (SD;range)
International Trauma Exposure Measure			
Traumatic events (ITEM; 0-66)	8.64 (6.64;1-22)	8.16 (5.24;1-24)	8.40 (5.93;1-24)
Traumatic life events (Crit.A; 0-48)	4.32 (3.40;0-11)	3.88 (2.60;0-10)	4.10 (3.01;0-11)
Psyc. threatening events (Crit.B; 0-18)	4.32 (3.61;0-12)	4.28 (3.58;0-14)	4.30 (3.56;0-14)
Developmental period			
Childhood events (0-12 years; 0-22)	2.44 (2.55;0-8)	1.64 (2.08;0-9)	2.04 (2.34;0-9)
Traumatic life events (Crit.A; 0-16)	1.04 (1.51;0-5)	0.92 (01.19;0-4)	0.98 (1.35;0-5)
Psyc. threatening events (Crit.B; 0-6)	1.40 (1.47;0-4)	0.72 (1.34;0-5)	1.06 (1.43;0-5)
Adolescent events (13-18 years; 0-22)	3.72 (3.23;0-9)	2.88 (2.42;0-10)	3.30 (2.86;0-10)
Traumatic life events (Crit.A; 0-16)	1.88 (1.79;0-5)	1.12 (1.17;0-5)	1.50 (1.54;0-5)
Psyc. threatening events (Crit.B; 0-6)	1.84 (1.84;0-5)	1.76 (1.61;0-5)	1.80 (1.71;0-5)
Adulthood events (19+ years; 0-22)	2.48 (2.24;0-9)	3.64 (2.94;0-9)	3.06 (2.65;0-9)
Traumatic life events (Crit.A; 0-16)	1.40 (1.35;0-5)	1.84 (1.86;0-6)	1.62 (1.63;0-6)
Psyc. threatening events (Crit.B; 0-6)	1.08 (1/15;0-4)	1.80 (1.80;0-6)	1.44 (1.54;0-6)

Note: For the International Trauma Exposure Measure (ITEM), Criterion A comprises 16 items describing events that are direct or indirect threat to life, or to physical or sexual safety; these reflect the DSM-5 definition of trauma exposure. Criterion B comprises 5 items describing events that are psychologically threatening and are considered traumatic in line with ICD-11 guidelines.

Table S3. Questionnaire and ecological momentary assessment (EMA) completion rates at 3-, 6- and 9-month follow-ups for participants in EMDR (+NPC; n = 25) and NPC (n = 25) conditions.

	EMDR+NPC<br>n (%)	NPC<br>n (%)	Total<br>n (%)
EMA completion			
Any at follow-up (≥1 day)			
3-month	20 (80.0)	22 (88.0)	42 (84.0)
6-month	22 (88.0)	18 (72.0)	40 (80.0)
9-month	18 (72.0)	16 (64.0)	34 (68.0)
≥5day at baseline/follow-up			
Baseline	24 (96.0)	24 (96.0)	48 (96.0)
3-month	18 (72.0)	21 (84.0)	39 (78.0)
6-month	22 (88.0)	16 (64.0)	38 (76.0)
9-month	12 (48.0)	12 (48.0)	24 (48.0)
Number of EMA days completed	Mean (SD)	Mean (SD)	Mean (SD)
Baseline	11.5 (2.6)	11.3 (3.3)	11.4 (2.9)
3-month	10.0 (3.9)	10.2 (3.3)	10.1 (3.6)
6-month	10.1 (3.0)	8.9 (3.8)	9.6 (3.4)
9-month	6.8 (4.6)	9.1 (4.7)	7.9 (4.7)
Questionnaire completion (follow-up)			
3-month	20 (80.0)	21 (84.0)	41 (82.0)
6-month	22 (88.0)	21 (84.0)	43 (86.0)
9-month	22 (88.0)	17 (68.0)	38 (76.0)

Note: IQR = interquartile range. At 3-month follow-up one participant completing measures only did so for the ITQ and MDI; at 6-month follow-up one participant completing measures did not respond on the MDI. Number of days completed (EMA) includes only those participants with EMA complete for at least one day at the relevant time point.

Table S4. Summary of therapy sessions for participants receiving EMDR (+NPC; n = 24). Values are frequencies (percentages) unless otherwise stated.

Therapy completion (of 24)	22 (91.7%)
Number of sessions (mean (SD; range))	13.0 (3.4; 6-16)
Mean length of session (minutes; mean (SD; range))	69.8 (6.3; 61.4,82.0)
Mean interval between sessions (days; mean (SD; range))	10.1 (1.6; 7.6-14.0)
Therapist	
A	13 (54.5)
B	11 (45.5)
Mode of therapy	
In-person	2 (8.3)
Online	18 (75.0)
In-person and online	4 (16.7)
Target memory category (in ≥1 session)	
FND-related memories and images	3 (12.5)
Non-FND distressing or traumatic memories	5 (20.8)
Both FND-related and non-FND distressing or traumatic memories/images	16 (66.7)
Stimulation type	
Bilateral tapping only	19 (79.2)
Eye movements only	3 (12.5)
Bilateral tapping and Eye movements	1 (4.2)
Bilateral tapping, Eye movements and Audio tones	1 (4.2)

One participant allocated to the EMDR+NPC condition withdrew prior to attending their first EMDR session so was not included in summary analyses of EMDR sessions. For 2 participants, the initial two sessions were in-person and all remaining sessions were online. One participant received therapy online for all sessions but one (in-person session 12 of 16) while one participant received therapy in-person for all sessions but one (online session 14 of 16). FND-related memories and images includes memories/images related to FND onset, FND symptoms or flashforward; Non-FND distressing or traumatic memories includes memories/images related to childhood trauma, adulthood trauma and beliefs; Target memory category classifications are not mutually exclusive to participants (i.e., across sessions participants typically accessed a number of Target memories that were classified under each headings).

Table S5. Adjusted descriptive statistics and between-group effect size estimates for ecological momentary assessment (EMA) measures of functional symptoms according to whether or not participants met the (ITQ) criteria for PTSD at baseline. symptom type (ITT approach).

	With PTSD			Without PTSD		
	EMDR+NPC	NPC	EMDR+NPC vs NPC	EMDR+NPC	NPC	EMDR+NPC vs NPC
	Mean (SE)	Mean (SE)	Effect size (CI)	Mean (SE)	Mean (SE)	Effect size (CI)
Seizure frequency						
Baseline	8.13 (3.22)	13.63 (3.31)		8.68 (3.02)	9.38 (2.84)	
3-month F-U	7.05 (3.68)	14.79 (3.44)	0.16 (-0.16,0.47)	0.61 (3.08)	9.13 (3.02)	0.71 (0.42,1.00)
6-month F-U	6.01 (3.26)	10.61 (3.70)	0.36 (-0.04,0.75)	0.63 (3.15)	7.41 (3.22)	0.67 (0.35,0.99)
9-month F-U	8.34 (3.37)	11.06 (4.12)	0.35 (-0.04,0.74)	2.29 (3.55)	11.75 (3.30)	0.62 (0.27,0.97)
Severity						
Baseline	3.93 (0.48)	2.50 (0.44)		3.52 (0.35)	5.65 (0.39)	
3-month F-U	3.83 (0.53)	2.08 (0.47)	-0.20 (-0.39,-0.001)	2.40 (0.38)	5.25 (0.41)	0.33 (0.18,0.48)
6-month F-U	2.80 (0.49)	2.41 (0.52)	0.64 (0.39,0.89)	2.48 (0.38)	5.02 (0.45)	0.19 (0.02,0.36)
9-month F-U	3.80 (0.52)	3.11 (0.61)	0.45 (0.21,0.69)	2.20 (0.46)	5.22 (0.45)	0.41 (0.22,0.59)
Interference						
Baseline	3.85 (0.50)	2.76 (0.46)		3.38 (0.36)	5.86 (0.40)	
3-month F-U	3.66 (0.55)	2.59 (0.49)	0.01 (-0.18,0.20)	2.10 (0.40)	5.33 (0.42)	0.36 (0.21,0.51)
6-month F-U	2.75 (0.51)	2.49 (0.54)	0.40 (0.15,0.64)	2.26 (0.39)	5.14 (0.46)	0.19 (0.02,0.36)
9-month F-U	3.79 (0.53)	3.30 (0.63)	0.28 (0.05,0.52)	1.97 (0.48)	5.17 (0.47)	0.35 (0.16,0.53)
Distress						
Baseline	4.09 (0.49)	2.24 (0.45)		3.36 (0.36)	5.64 (0.40)	
3-month F-U	3.98 (0.55)	2.47 (0.49)	0.15 (-0.05,0.34)	2.04 (0.39)	5.06 (0.42)	0.34 (0.19,0.49)
6-month F-U	2.86 (0.50)	2.33 (0.53)	0.57 (0.32,0.82)	2.08 (0.39)	5.10 (0.45)	0.34 (0.17,0.51)
9-month F-U	3.98 (0.53)	3.18 (0.62)	0.46 (0.22,0.69)	2.04 (0.47)	4.93 (0.46)	0.28 (0.09,0.46)
PreOccupation						
Baseline	4.23 (0.50)	3.03 (0.46)		3.33 (0.36)	6.04 (0.40)	
3-month F-U	4.11 (0.55)	2.75 (0.49)	-0.07 (-0.26,0.13)	1.82 (0.40)	5.51 (0.42)	0.45 (0.30,0.60)
6-month F-U	2.77 (0.50)	2.99 (0.54)	0.60 (0.35,0.85)	2.13 (0.39)	5.65 (0.46)	0.37 (0.20,0.54)
9-month F-U	3.60 (0.53)	3.48 (0.63)	0.46 (0.22,0.70)	2.42 (0.48)	5.49 (0.47)	0.16 (-0.02,0.35)

Note: Three participants did not indicate a second FND symptom and, as such, did not provide any EMA ratings. Effect sizes = Hedge's g; Positive effect sizes represent an improvement in symptoms that is greater in EMDR+NPC relative to NPC. For seizure frequency, effect sizes were calculated from values that were transformed – raw mean (SE) values are shown for ease of interpretation.

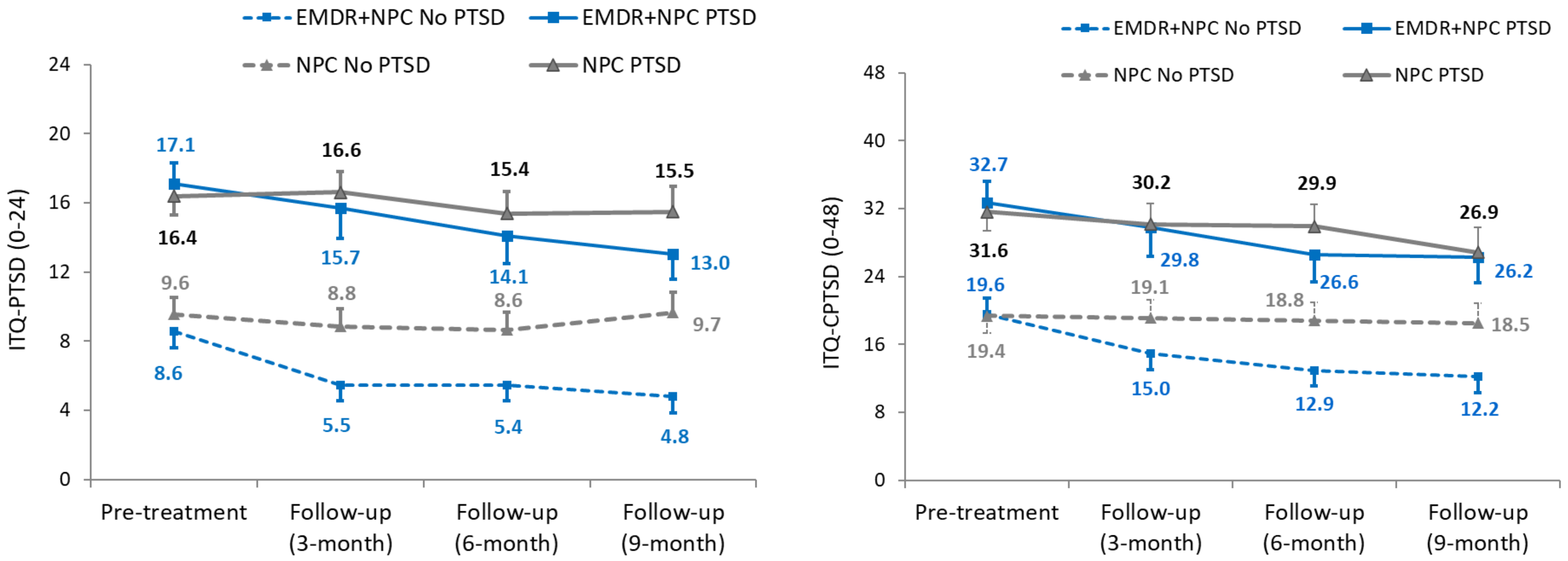

Figure S1a and S1b. International Trauma Questionnaire (ITQ)-PTSD (S1a) and CPTSD (S1b) for participants in EMDR (+NPC) and NPC conditions at screening and follow-up periods (ITT approach) according to whether or not participants met the (ITQ) criteria for PTSD at baseline. Data labels are adjusted mean values. Error bars represent standard errors. At 3-, 6- and 9-month follow, respective between-group Hedge's *g* (CI) for ITQ-PTSD scores were 0.47 (-0.39,1.32), 0.58 (-0.28,1.44) and 0.90 (0.01,1.79) for participants meeting criteria for PTSD and 0.47 (-0.24,1.17), 0.43 (-0.27,1.14) and 0.76 (0.03,1.48) for participants not meeting criteria for PTSD. At 3-, 6- and 9-month follow, respective between-group Hedge's *g* (CI) for ITQ-CPTSD scores were 0.17 (-0.67,1.02), 0.53 (-0.33,1.39) and 0.21 (-0.64,1.05) for participants meeting criteria for PTSD and 0.44 (-0.26,1.15), 0.62 (-0.10,1.33) and 0.66 (-0.06,1.38) for participants not meeting criteria for PTSD.

Table S6. Adjusted descriptive statistics and between-group effect size estimates for . International Trauma Questionnaire (ITQ)-PTSD and CPTSD scores according to whether or not participants experience functional seizures (ITT approach).

	FND with Seizure			FND without Seizure		
	EMDR+NPC	NPC	EMDR+NPC vs NPC	EMDR+NPC	NPC	EMDR+NPC vs NPC
	Mean (SE)	Mean (SE)	Effect size (CI)	Mean (SE)	Mean (SE)	Effect size (CI)
ITQ PTSD (0-24)						
Baseline	12.06 (1.47)	13.44 (1.47)		10.89 (1.95)	11.00 (1.95)	
3-month F-U	6.81 (1.57)	13.42 (1.52)	0.78 (0.08,1.48)	9.84 (2.09)	11.01 (2.09)	0.19 (-0.70,1.07)
6-month F-U	7.03 (1.52)	12.23 (1.53)	0.57 (-0.12,1.26)	7.94 (2.04)	9.59 (2.12)	0.27 (-0.61,1.16)
9-month F-U	6.35 (1.53)	12.08 (1.60)	0.65 (-0.05,1.34)	8.44 (2.04)	13.92 (2.55)	0.95 (0.01,1.88)
ITQ CPTSD (0-24)						
Baseline	24.88 (2.77)	26.63 (2.77)		23.22 (3.70)	21.44 (3.70)	
3-month F-U	17.17 (2.96)	25.14 (2.86)	0.50 (-0.18,1.19)	21.35 (3.92)	23.35 (3.92)	0.34 (-0.55,1.22)
6-month F-U	14.68 (2.87)	24.90 (2.88)	0.69 (-0.01,1.38)	18.55 (3.84)	20.40 (3.98)	0.32 (-0.56,1.21)
9-month F-U	14.02 (2.89)	21.96 (3.02)	0.50 (-0.19,1.19)	20.12 (3.85)	26.40 (4.74)	0.72 (-0.19,1.63)

Note: Three participants did not indicate a second FND symptom and, as such, did not provide any EMA ratings. Effect sizes = Hedge’s g; Positive effect sizes represent an improvement in symptoms that is greater in EMDR+NPC relative to NPC

Table S7. Adjusted descriptive statistics and between-group effect size estimates for ecological momentary assessment (EMA) measures of functional symptoms (per-protocol approach).

	EMDR+NPC	NPC	EMDR+NPC vs NPC
	Mean (SE)	Mean (SE)	Effect size (95% CI)
Frequency (per week)			
Seizures			
Baseline	9.54 (2.41)	11.39 (2.24)	
3-month Follow-up	3.30 (2.62)	11.28 (2.38)	0.57 (0.35,0.78)
6-month Follow-up	3.74 (2.43)	8.85 (2.60)	0.53 (0.29,0.78)
9-month Follow-up	5.47 (2.73)	11.66 (2.91)	0.45 (0.19,0.71)
Frequency (% of days)			
Seizures			
Baseline	43.35 (16.95)	53.88 (16.30)	
3-month Follow-up	13.73 (8.97)	48.88 (17.56)	0.60 (0.39,0.82)
6-month Follow-up	7.03 (4.82)	53.05 (18.61)	0.77 (0.52,1.03)
9-month Follow-up	17.72 (11.45)	61.45 (20.54)	0.95 (0.68,1.22)

Note: Effect sizes = Hedge's g; Positive effect sizes represent an improvement in symptoms that is greater in EMDR+NPC relative to NPC. For seizure frequency, effect sizes were calculated from values that were transformed – raw mean (SE) values are shown for ease of interpretation.

Table S8. Adjusted descriptive statistics and between-group effect size estimates for ecological momentary assessment (EMA) measures of functional symptoms (per-protocol approach).

	EMDR+NPC	NPC	EMDR+NPC vs NPC
	Mean (SE)	Mean (SE)	Effect size (95% CI)
Overall (0-10)			
Severity			
Baseline	3.85 (0.33)	4.33 (0.32)	
3-month Follow-up	2.92 (0.35)	3.90 (0.34)	0.23 (0.11,0.35)
6-month Follow-up	2.71 (0.33)	3.94 (0.38)	0.35 (0.21,0.49)
9-month Follow-up	3.02 (0.41)	4.14 (0.43)	0.30 (0.15,0.45)
Interference			
Baseline	3.72 (0.34)	4.59 (0.32)	
3-month Follow-up	2.77 (0.36)	4.20 (0.35)	0.25 (0.13,0.38)
6-month Follow-up	2.57 (0.34)	4.07 (0.39)	0.29 (0.15,0.43)
9-month Follow-up	2.90 (0.42)	4.24 (0.44)	0.21 (0.06,0.36)
Distress			
Baseline	3.78 (0.33)	4.18 (0.33)	
3-month Follow-up	2.81 (0.36)	3.96 (0.35)	0.31 (0.19,0.44)
6-month Follow-up	2.47 (0.34)	3.86 (0.38)	0.41 (0.27,0.55)
9-month Follow-up	3.10 (0.42)	4.09 (0.44)	0.25 (0.10,0.39)
PreOccupation			
Baseline	3.79 (0.34)	4.75 (0.33)	
3-month Follow-up	2.64 (0.36)	4.29 (0.35)	0.28 (0.16,0.40)
6-month Follow-up	2.43 (0.34)	4.52 (0.39)	0.46 (0.32,0.60)
9-month Follow-up	3.16 (0.42)	4.47 (0.44)	0.14 (-0.01,0.29)

Note: Three participants did not indicate a second FND symptom and, as such, did not provide any VAS ratings. Effect sizes = Hedge's g; Positive effect sizes represent an improvement in symptoms that is greater in EMDR+NPC relative to NPC

Table S9. Adjusted descriptive statistics and between-group effect size estimates for ecological momentary assessment (EMA) measures of functional symptoms according to symptom type (per-protocol approach).

	Functional Seizures			Functional Motor Symptoms			Functional Cognitive Symptoms		
	EMDR+NPC	NPC	EMDR+NPC vs NPC	EMDR+NPC	NPC	EMDR+NPC vs NPC	EMDR+NPC	NPC	EMDR+NPC vs NPC
	Mean (SE)	Mean (SE)	Effect size (95% CI)	Mean (SE)	Mean (SE)	Effect size (95% CI)	Mean (SE)	Mean (SE)	Effect size (95% CI)
Severity									
Baseline	2.84 (0.43)	3.21 (0.42)		3.97 (0.37)	5.19 (0.35)		4.74 (0.53)	4.59 (0.59)	
3-month F-U	1.65 (0.47)	2.70 (0.45)	0.36 (0.14,0.58)	3.33 (0.39)	4.69 (0.37)	0.06 (-0.11,0.23)	3.80 (0.57)	4.32 (0.61)	0.31 (-0.002,0.62)
6-month F-U	1.70 (0.43)	2.21 (0.49)	0.07 (-0.18,0.33)	2.64 (0.38)	4.70 (0.41)	0.35 (0.16,0.55)	3.77 (0.54)	4.90 (0.70)	0.59 (0.22,0.95)
9-month F-U	2.36 (0.50)	2.18 (0.54)	-0.29 (-0.55,-0.02)	3.10 (0.45)	4.93 (0.44)	0.26 (0.06,0.46)	3.59 (0.72)	5.32 (0.84)	0.86 (0.43,1.29)
Interference									
Baseline	2.69 (0.44)	3.89 (0.44)		3.74 (0.38)	5.11 (0.36)		4.73 (0.54)	4.77 (0.60)	
3-month F-U	1.62 (0.48)	3.27 (0.46)	0.22 (-0.001,0.43)	2.93 (0.41)	4.77 (0.38)	0.20 (0.03,0.37)	3.76 (0.58)	4.57 (0.63)	0.37 (0.06,0.69)
6-month F-U	1.60 (0.45)	2.71 (0.50)	-0.04 (-0.30,0.21)	2.29 (0.39)	4.86 (0.42)	0.51 (0.32,0.71)	3.81 (0.55)	4.63 (0.71)	0.38 (0.02,0.74)
9-month F-U	2.28 (0.51)	2.57 (0.55)	-0.44 (-0.71,-0.18)	2.87 (0.46)	4.92 (0.45)	0.29 (0.09,0.49)	3.56 (0.72)	5.22 (0.83)	0.79 (0.37,1.22)
Distress									
Baseline	3.21 (0.44)	4.09 (0.43)		3.66 (0.38)	4.61 (0.36)		4.46 (0.53)	3.84 (0.60)	
3-month F-U	2.18 (0.47)	3.25 (0.46)	0.08 (-0.13,0.30)	2.94 (0.40)	4.58 (0.38)	0.27 (0.11,0.44)	3.31 (0.58)	4.04 (0.62)	0.60 (0.28,0.91)
6-month F-U	1.83 (0.44)	2.71 (0.49)	-0.002 (-0.26,0.25)	2.18 (0.38)	4.39 (0.41)	0.49 (0.30,0.69)	3.39 (0.55)	4.49 (0.70)	0.75 (0.38,1.12)
9-month F-U	2.47 (0.51)	2.77 (0.54)	-0.24 (-0.51,0.02)	2.80 (0.46)	4.73 (0.45)	0.39 (0.19,0.59)	4.03 (0.72)	4.78 (0.83)	0.60 (0.18,1.02)
PreOccupation									
Baseline	3.37 (0.44)	5.46 (0.44)		3.63 (0.38)	4.86 (0.36)		4.37 (0.54)	3.94 (0.60)	
3-month F-U	2.06 (0.48)	4.05 (0.46)	-0.04 (-0.26,0.17)	2.76 (0.41)	4.58 (0.38)	0.22 (0.05,0.39)	3.10 (0.58)	4.25 (0.63)	0.69 (0.37,1.01)
6-month F-U	1.84 (0.45)	4.16 (0.50)	0.09 (-0.17,0.34)	2.09 (0.39)	4.66 (0.42)	0.50 (0.30,0.69)	3.37 (0.55)	4.75 (0.71)	0.79 (0.41,1.16)
9-month F-U	2.51 (0.51)	3.72 (0.55)	-0.35 (-0.61,-0.08)	3.12 (0.46)	4.90 (0.45)	0.21 (0.01,0.41)	3.85 (0.73)	4.79 (0.84)	0.60 (0.18,1.02)

Note: Three participants did not indicate a second FND symptom and, as such, did not provide any EMA ratings. Effect sizes = Hedge's g; Positive effect sizes represent an improvement in symptoms that is greater in EMDR+NPC relative to NPC

Table S10. Descriptive statistics and (adjusted) between-group effect size estimates for standardised questionnaire outcome measures (per-protocol approach).

	EMDR+NPC	NPC	EMDR+NPC vs NPC
	Mean (SE)	Mean (SE)	Effect size (95% CI)
PTSD			
ITQ PTSD Symptom Score (0-24)			
Baseline	12.36 (6.28)	12.56 (5.95)	
3-month Follow-up	7.61 (5.16)	11.95 (5.73)	0.74 (0.10,1.38)
6-month Follow-up	7.57 (5.68)	11.10 (5.49)	0.59 (-0.02,1.21)
9-month Follow-up	7.29 (5.86)	11.81 (4.61)	0.75 (0.09,1.41)
ITQ DSO Symptom Score (0-24)			
Baseline	12.95 (5.53)	12.20 (6.28)	
3-month Follow-up	10.06 (4.50)	11.33 (5.99)	0.27 (-0.35,0.89)
6-month Follow-up	8.43 (5.75)	11.48 (5.75)	0.63 (0.02,1.24)
9-month Follow-up	9.14 (6.17)	9.38 (5.46)	0.17 (-0.47,0.81)
ITQ CPTSD Symptom Score (0-48)			
Baseline	25.32 (11.17)	24.76 (11.34)	
3-month Follow-up	17.67 (8.49)	23.29 (10.64)	0.53 (-0.09,1.16)
6-month Follow-up	16.00 (10.72)	22.57 (10.44)	0.67 (0.06,1.28)
9-month Follow-up	16.43 (11.48)	21.19 (8.72)	0.52 (-0.13,1.16)
Depression / Anxiety			
PHQ-9 (0-27)			
Baseline	14.82 (3.95)	14.24 (4.38)	
3-month Follow-up	10.88 (4.31)	12.67 (5.15)	0.61 (-0.03,1.25)
6-month Follow-up	11.14 (5.21)	12.62 (4.70)	0.49 (-0.11,1.09)
9-month Follow-up	11.33 (6.22)	11.44 (4.90)	0.14 (-0.50,0.78)
GAD-7 (0-21)			
Baseline	12.36 (5.15)	11.04 (4.93)	
3-month Follow-up	8.76 (5.26)	9.86 (5.70)	0.30 (-0.33,0.93)
6-month Follow-up	8.00 (6.49)	9.57 (4.88)	0.51 (-0.09,1.12)
9-month Follow-up	8.52 (5.94)	8.88 (4.21)	0.19 (-0.45,0.82)
Dissociation			
MDI-Total (30-150)			
Screening	75.41 (24.60)	71.64 (22.60)	
3-month Follow-up	66.26 (26.41)	65.57 (21.13)	0.31 (-0.30,0.92)
6-month Follow-up	58.38 (19.70)	66.80 (23.27)	0.47 (-0.14,1.08)
9-month Follow-up	59.81 (21.12)	63.63 (22.95)	0.33 (-0.31,0.97)

<u>HRQoL/Functioning</u>			
WHODAS 2.0 Total (0-100)			
Baseline	46.07 (91.12)	53.39 (19.26)	
3-month Follow-up	37.60 (14.94)	49.32 (20.57)	0.33 (-0.30,0.96)
6-month Follow-up	35.18 (19.35)	49.66 (15.18)	0.57 (-0.03,1.18)
9-month Follow-up	37.45 (19.35)	47.17 (18.00)	0.32 (-0.32,0.96)
EQ-5D-5L Value (0-100)			
Baseline	0.621 (0.230)	0.497 (0.209)	
3-month Follow-up	0.714 (0.239)	0.467 (0.273)	0.58 (-0.06,1.22)
6-month Follow-up	0.646 (0.292)	0.507 (0.226)	0.30 (-0.30,0.90)
9-month Follow-up	0.636 (0.268)	0.548 (0.229)	0.04 (-0.60,0.68)
EQ-VAS (0-100)			
Baseline	49.05 (18.91)	44.72 (18.47)	
3-month Follow-up	52.47 (21.84)	43.90 (17.88)	0.50 (-0.14,1.14)
6-month Follow-up	58.38 (23.29)	42.76 (16.83)	0.77 (0.15,1.39)
9-month Follow-up	50.86 (21.20)	53.00 (15.76)	-0.14 (-0.78,0.50)

Note: MDI = Multiscale Dissociation Inventory; PTSD = Post-Traumatic Stress Disorder; ITQ = International Trauma Questionnaire; DSO = Disturbances in Self-Organization; CPTSD = Complex Post-Traumatic Stress Disorder; HRQoL = Health-related quality of life; WHODAS 2.0 = WHO Disability Assessment Schedule version 2.0. WHODAS 2.0 mean (SD) values were calculated using complex scoring, based on item response theory-based scoring (which accounts for multiple levels of difficulty for each WHODAS item; Üstün, 2010): EQ-5D-5L = EQ-5D-5L health state evaluation; EQ-VAS = current overall health rating (today); PHQ-9 = Patient Health Questionnaire-9; GAD-7 = Generalized Anxiety Disorder-7. Effect sizes = Hedge's g; Positive effect sizes represent an improvement in symptoms that is greater in EMDR+NPC relative to NPC. For EQ-5D-5L, effect sizes were calculated from values that were transformed – raw mean (SE) values are shown for ease of interpretation.
